# Extended Kalman Filters, Least Squares Methods, and Successive Linear Estimator for Inverse Problems in Groundwater Flow

**DOI:** 10.3390/e28101053

**Published:** 2026-09-25

**Authors:** Jun Li, Yonghong Hao, Qi Wang

**Affiliations:** 1 School of Mathematical Sciences, Tianjin Normal University, Tianjin 300387, China; nkjunli@foxmail.com; 2 Institute of Mathematics and Interdisciplinary Sciences, Tianjin Normal University, Tianjin 300387, China; 3 Tianjin Key Laboratory of Water Resources and Environment, Tianjin Normal University, Tianjin 300387, China; 4 Department of Mathematics, University of South Carolina, Columbia, SC 29208, USA

**Keywords:** hydraulic conductivity, ground water, Kalman filters, Richards equation, least squares methods, successive linear estimator

## Abstract

We formulate the problem of estimating hydraulic conductivity from measured hydraulic head data in groundwater flow within the unified framework of parameter estimation using the extended Kalman filter (EKF) and least-squares optimization. We first formulate the EKF and its variants as problems of least-squares optimization. We then show that the successive linear estimator can be interpreted as a variant of the EKF, with its initial estimate obtained through co-Kriging. Furthermore, by showing that the EKF is equivalent to a one-step Newton iteration for solving a least-squares problem, we reformulate the hydraulic conductivity estimation problem as an associated least-squares optimization problem. Finally, we present an algorithm for estimating hydraulic conductivity from unsaturated soil data.

## 1. Introduction

Reliable characterization of hydraulic conductivity is a central task in groundwater hydrology and subsurface flow modeling. Hydraulic conductivity controls the spatial distribution of hydraulic head, Darcy flux, solute transport pathways, and travel times, but is rarely observed directly at the spatial resolution required by predictive groundwater models. Field measurements of conductivity are typically sparse, indirect, and scale-dependent, whereas hydraulic head observations are more readily available. However, the hydraulic head value depends on conductivity through a nonlinear forward model, known as the Richards equation. As a result, the reconstruction of hydraulic conductivity from hydraulic head data is a classical inverse problem in hydrogeology, combining partial differential equation models, spatial statistics, optimization, and uncertainty quantification [1,2,3,4,5,6,7]. 

In saturated aquifers, the hydraulic head is commonly modeled by the steady groundwater flow equation, i.e., the saturated form of the Richards equation in the steady state, into which the hydraulic conductivity field enters as a spatially heterogeneous coefficient. Since conductivity is positive and often varies over several orders of magnitude, it is standard to represent uncertainty about the logarithmic conductivity or logarithmic hydraulic conductivity by a random field prior. In unsaturated soil, however, the inverse problem is more complex because the hydraulic conductivity depends on the water content or pressure head through constitutive relationships. In this case, the Richards equation couples pressure head, water content, and hydraulic conductivity in a nonlinear and often strongly degenerate manner [8,9,10,11,12,13]. These features make identification of hydraulic conductivity in both saturated and unsaturated soils ill-posed, sensitive to measurement error, and dependent on appropriate prior information. In summary, the saturated flow problem in Section 3 is steady-state, while the unsaturated flow problem in Section 4 is transient. Together with the saturated/unsaturated nature of the medium, this distinction forms the basis for the different inversion strategies presented in this work. 

A class of approaches for hydrogeological inverse problems uses geostatistical priors to describe spatial variability and regularize the estimation problem. Kriging and co-Kriging provide the best linear unbiased estimators based on covariance and cross-covariance information, allowing direct conductivity measurements and auxiliary hydraulic head observations to be incorporated within a unified spatial estimator [6,14,15,16,17,18,19,20]. In this setting, the prior mean and covariance of the logarithmic conductivity field encode both geological structure and measurement information. Such priors are particularly useful in inverse problems, as they reduce non-uniqueness and provide a statistically meaningful initial estimate for iterative reconstruction methods. 

Among iterative stochastic inverse methods, the successive linear estimator (SLE) introduced by [21] has been influential for estimating conditional effective hydraulic conductivity and hydraulic head fields [22,23,24,25,26,27,28,29,30,31]. The essential idea is to begin with a geostatistical estimate of the log-conductivity field, then update it successively using discrepancies between measured and simulated hydraulic heads. At each iteration, the nonlinear map from conductivity to hydraulic head is linearized and the update is computed from covariance and cross-covariance relationships between parameter perturbations and head perturbations. The resulting formula has the structure of a gain-times-innovation correction in which the current estimate is adjusted by a linear combination of hydraulic head residuals. This structure suggests a close connection between SLE and recursive filtering methods. 

Kalman filtering provides a natural mathematical framework for such recursive estimation. For linear dynamical systems with quadratic loss functions, the Kalman filter gives the optimal linear unbiased update and can be derived equivalently from minimum-variance estimation, Bayesian Gaussian conditioning, or recursive least-squares optimization [32,33,34,35,36]. For nonlinear systems, the extended Kalman filter (EKF) applies the same prediction correction mechanism after local linearization of the nonlinear state and observation maps [37,38]. In static parameter estimation problems, the parameter can be treated as a state with identity dynamics, with the observation operator as the nonlinear forward map from parameters to measured quantities. This viewpoint has been widely used in subsurface data assimilation and groundwater model calibration, including ensemble and sequential extensions for joint state parameter estimation [39,40,41,42,43,44]. 

The purpose of this paper is to establish the connection between SLE and EKF explicitly in the setting of hydraulic conductivity estimation. We formulate the successive linear estimation of the hydraulic conductivity from measured hydraulic head data as a parameter estimation problem within the framework of extended Kalman filtering and least-squares optimization. First, we review the Kalman filter and the EKF from the perspective of weighted least-squares minimization. In particular, Kalman correction can be interpreted as a one-step Newton update applied to a quadratic least-squares objective, while the EKF corresponds to applying this same update after linearizing the nonlinear observation operator. This least-squares viewpoint clarifies the roles of the prior covariance, observation covariance, sensitivity matrix, and innovation term. 

Building on this formulation, the methods presented in this work are applicable to a range of hydrogeological inverse problems, including steady-state confined aquifer characterization via hydraulic tomography, transient parameter estimation in the vadose zone, and joint inversion of hydraulic conductivity and hydraulic head observations. The previous literature has demonstrated that SLE can recover transmissivity fields from head measurements in confined aquifers [21,25,27], and EKF/EnKF workflows have been widely adopted for transient groundwater data assimilation [40,41]. This paper develops a unified theoretical framework connecting SLE and EKF, then extends the methodology to variably saturated Richards flow. Rather than presenting numerical or field demonstrations of algorithm performance, the derived consistency relations provide a rigorous mathematical foundation for these common hydrogeological inversion workflows. 

The contributions of this work are summarized as follows. 

First, we establish a rigorous mathematical equivalence between the successive linear estimator (SLE) and the extended Kalman filter (EKF) for hydraulic conductivity estimation. Although both methods are widely used in hydrogeological inversion, their formal connection has not been explicitly demonstrated in the previous literature. We show that SLE can be reinterpreted as an EKF-type correction step with a geostatistical initialization and that the associated SLE update corresponds precisely to a one-step Newton iteration of a least-squares optimization problem. 

Second, we reformulate the Kalman filter and EKF from a unified least-squares perspective, providing a common theoretical basis that clarifies the roles of prior covariance, observation covariance, sensitivity matrix, and innovation term in both sequential and iterative estimation frameworks. 

Third, we extend the unified SLE–EKF formulation to transient unsaturated flow governed by the Richards equation. Two complementary strategies are developed: a parametric EKF approach with basis expansion, and an ensemble Kalman filter (EnKF) approach that permits data-driven deviations from the van Genuchten–Mualem model. This extension provides a systematic framework for conductivity estimation in the vadose zone, where the traditional SLE formulation was previously limited to saturated steady-state conditions.

## 2. Kalman Filters and Least Squares Methods

In this section, we review the standard Kalman filter and the extended Kalman filter as background material for the subsequent developments. These formulations are well established in the literature, and are presented here to set up the notation and facilitate the least-squares reinterpretation that follows in later sections.

### 2.1. Kalman Filters for Discrete Linear Systems

The Kalman filter is an optimal linear state estimation algorithm in the context of unbiased estimators. It fuses a prediction model with observation data to estimate the true state of a dynamic system in the presence of noise and uncertainty [32]. We use the following discrete linear time-invariant (LTI) system as an example:

(1) $$\begin{array}{c} \begin{array}{c} \begin{array}{cc} \mathrm{state}\;\mathrm{equation}:\;x_{k} & =Ax_{k-1}+Bu_{k}+w_{k},\\ \mathrm{observational}\;\mathrm{equation}:\;z_{k} & =Hx_{k}+v_{k},\;k=1,\cdots , \end{array} \end{array} \end{array}$$

where $x_{k}\in R^{n\times 1}$ denotes the state vector of the dynamic system at time step *k*, which is the true *n*-dimensional state to be estimated; $A\in R^{n\times n}$ serves as the state transition matrix, illustrating how the state evolves from time step k−1 to *k* in the absence of control inputs and process noise, $w_{k}$, while $B\in R^{n\times m}$ is the control input matrix mapping the *m*-dimensional control input $u_{k}\in R^{m\times 1}$ to its impact on the state transition in $x_{k-1}\to x_{k}$ in $R^{n}$ and $z_{k}\in R^{p\times 1}$ represents the observation (measurement) vector at time step *k* acquired from sensors or other measurement devices, where *p* is the number of observations. In addition, $H\in R^{p\times n}$ is the observation matrix establishing the relationship between the *n*-dimensional system state $x_{k}$ and the *p*-dimensional observation $z_{k}$, $w_{k}\in R^{n\times 1}∼N\left(0,Q_{k}\right)$ is the process noise with a zero mean and covariance $Q_{k}=E\left[w_{k}w_{k}^{T}\right]\in R^{n\times n}$, and $v_{k}\in R^{p\times 1}∼N\left(0,R_{k}\right)$ is the measurement noise with a zero mean and covariance $R_{k}=E\left[v_{k}v_{k}^{T}\right]\in R^{p\times p}$. Finally, $w_{k}$ and $v_{k}$ are assumed to be independent Gaussian white noise, and are uncorrelated with each other.

We note that while *n* and *m* are independent positive integers, *n*, *m*, and *p* are not completely independent: if p+m<n, i.e., the number of control variables and measurements is smaller than the dimension of the state vector to be estimated, then problems could be encountered in terms of properly identifying the state. In fact, the state estimation problem of system (1) may not have a solution or unique solution of $x_{k}$, given $x_{k-1},u_{k},z_{k},w_{k},v_{k}$; we also need to supply an initial state

(2) $$\begin{array}{c} x_{0}=x_{0}^{-}+v_{0}, \end{array}$$

where $x_{0}^{-}$ is a given initial value and $v_{0}$ is the noise.

Let ${\hat{x}}_{k}^{-}$ denote the a priori state estimate at time *k*, i.e., the prediction before incorporating the observation $z_{k}$, which will be specified later. Given ${\hat{x}}^{-}$, the posterior estimate at time *k* is

(3) $$\begin{array}{c} \begin{array}{c} {\hat{x}}_{k}={\hat{x}}_{k}^{-}+K_{k}\left(z_{k}-H{\hat{x}}_{k}^{-}\right), \end{array} \end{array}$$

where $K_{k}$ is the Kalman gain given below. The objective is to find the Kalman gain matrix $K_{k}$ such that the estimate ${\hat{x}}_{k}$ approaches the true state $x_{k}$ as an unbiased estimator, i.e., $E\left[x_{k}\right]=E\left[{\hat{x}}_{k}\right]$. 

Define the estimation error as

(4) $$\begin{array}{c} \begin{array}{c} e_{k}=x_{k}-{\hat{x}}_{k}\\ =x_{k}-{\hat{x}}_{k}^{-}-K_{k}\left(Hx_{k}+v_{k}-H{\hat{x}}_{k}^{-}\right)\\ =\left(I-K_{k}H\right)\left(x_{k}-{\hat{x}}_{k}^{-}\right)-K_{k}v_{k}\\ =\left(I-K_{k}H\right)e_{k}^{-}-K_{k}v_{k}, \end{array} \end{array}$$

where $e_{k}^{-}=x_{k}-{\hat{x}}_{k}^{-}$ and I denotes an identity matrix of appropriate dimension. Then, $E[e_{k}]=0$ and

(5) $$\begin{array}{c} \begin{array}{c} E\left[e_{k}e_{k}^{T}\right]=E\left[\left(\left(I-K_{k}H\right)e_{k}^{-}-K_{k}v_{k}\right){\left(\left(I-K_{k}H\right)e_{k}^{-}-K_{k}v_{k}\right)}^{T}\right]\\ =\left(I-K_{k}H\right)E\left[e_{k}^{-}e_{k}^{-T}\right]{\left(I-K_{k}H\right)}^{T}+K_{k}E\left[v_{k}v_{k}^{T}\right]K_{k}^{T}\\ =\left(I-K_{k}H\right)P_{k}^{-}{\left(I-K_{k}H\right)}^{T}+K_{k}R_{k}K_{k}^{T}, \end{array} \end{array}$$

where

$$P_{k}^{-}=E\left[e_{k}^{-}e_{k}^{-T}\right],\;e_{k}^{-}=x_{k}-{\hat{x}}_{k}^{-}.$$

The objective is to minimize the trace of the expected error covariance matrix

$$\min_{K_{k}}\;\mathrm{tr}\left(E\left[e_{k}e_{k}^{T}\right]\right)$$

which leads to the optimal gain $K_{k}$.

(6) $$\begin{array}{c} \frac{\partial }{\partial K_{k}}\mathrm{tr}\left(E\left[e_{k}e_{k}^{T}\right]\right)=2\left(K_{k}HP_{k}^{-}H^{T}+K_{k}R_{k}\right)-2P_{k}^{-}H^{T}=0. \end{array}$$

This implies

(7) $$\begin{array}{c} K_{k}=P_{k}^{-}H^{T}{\left(HP_{k}^{-}H^{T}+R_{k}\right)}^{-1}, \end{array}$$

which is the Kalman gain. At this value,

(8) $$\begin{array}{c} \begin{array}{c} E\left[e_{k}e_{k}^{T}\right]=\left(I-K_{k}H\right)P_{k}^{-}{\left(I-K_{k}H\right)}^{T}+K_{k}R_{k}K_{k}^{T}\\ =\left(I-K_{k}H\right)P_{k}^{-}, \end{array} \end{array}$$

with $P_{k}^{-}$ as the a priori estimation error covariance matrix. Here, we define

(9) $$\begin{array}{c} P_{k}=E\left[e_{k}e_{k}^{T}\right]=\left(I-K_{k}H\right)P_{k}^{-}. \end{array}$$

Since we want the guess to be close to the state, we use

(10) $$\begin{array}{c} {\hat{x}}_{k}^{-}=A{\hat{x}}_{k-1}+Bu_{k}, \end{array}$$

(11) $$\begin{array}{c} e_{k}^{-}=x_{k}-{\hat{x}}_{k}^{-}=Ae_{k-1}+w_{k}. \end{array}$$

With this choice, we have a formula for $P_{k}^{-}$,

(12) $$\begin{array}{cc} P_{k}^{-} & =AP_{k-1}A^{\;T}+Q_{k}. \end{array}$$

The Kalman filter can be written into a prediction-correction scheme as follows (see Algorithm 1).

**Algorithm** **1** (Kalman Filter)**.** *Given an initial estimate ${\hat{x}}_{0}$ and initial error covariance $P_{0}$, the Kalman filter proceeds in the following way for each time step k=1,2,…*

*(1)*  *Prediction.*

*Given the posterior estimate ${\hat{x}}_{k-1}$ and covariance $P_{k-1}$, the prediction step computes the following.*

(13) $$\begin{array}{cc} a\;\mathrm{priori}\;\mathrm{state}\;\mathrm{estimate}\;{\hat{x}}_{k}^{-} & =A{\hat{x}}_{k-1}+Bu_{k} \end{array}$$

(14) $$\begin{array}{cc} a\;\mathrm{priori}\;\mathrm{error}\;\mathrm{covariance}\;P_{k}^{-} & =AP_{k-1}A^{\;T}+Q_{k} \end{array}$$

*(2)*  *Correction (Update).*

(15) $$\begin{array}{cc} \;\;\;\;\;\;\;\;\;\;\mathrm{Kalman}\;\mathrm{gain}:\;K_{k} & =P_{k}^{-}H^{\;T}\;{\left(HP_{k}^{-}H^{\;T}+R_{k}\right)}^{-1} \end{array}$$

(16) $$\begin{array}{cc} \;\;\mathrm{measurement}\;\mathrm{residual}:\;y_{k} & =z_{k}-H{\hat{x}}_{k}^{-} \end{array}$$

(17) $$\begin{array}{cc} a\;\mathrm{posteriori}\;\mathrm{state}\;\mathrm{estimate}:\;{\hat{x}}_{k} & ={\hat{x}}_{k}^{-}+K_{k}y_{k} \end{array}$$

(18) $$\begin{array}{cc} a\;\mathrm{posteriori}\;\mathrm{error}\;\mathrm{covariance}:\;P_{k} & =\left(I-K_{k}H\right)P_{k}^{-} \end{array}$$

From this point, we present a new derivation that connects the Kalman filter to least-squares optimization.

**Lemma** **1.** *Given $q\left(x\right)=q_{1}\left(x\right)+q_{2}\left(x\right)$, where $q_{1}$ and $q_{2}$ are quadratic functions,*

(19) $$\begin{array}{c} x_{m}=\arg\min_{x}q\left(x\right)=x^{*}-{\left(\nabla^{2}q\left(x^{*}\right)\right)}^{-1}\nabla q\left(x^{*}\right) \end{array}$$

*for any $x^{*}$. If we choose $x^{*}$ such that $\nabla q_{1}\left(x^{*}\right)=0$,*

(20) $$\begin{array}{c} x_{m}=\arg\min_{x}q\left(x\right)=x^{*}-{\left(\nabla^{2}q\left(x^{*}\right)\right)}^{-1}\nabla q_{2}\left(x^{*}\right). \end{array}$$

*This means that we can first optimize $q_{1}\left(x\right)$. The overall optimization can be obtained using the above one-step Newton’s formula. In general,*

(21) $$\begin{array}{c} Q_{m}\left(x\right)=\sum_{i=1}^{m}q_{i}\left(x\right), \end{array}$$

*where $q_{i}\left(x\right)$ are quadratic functions. The optimizer of $Q_{m}\left(x\right)$ can be obtained sequentially from the optimizer of $Q_{m-1}\left(x\right)$, $x_{m-1}$ by*

(22) $$\begin{array}{c} x_{m}=x_{m-1}-{\left[\nabla^{2}Q_{m}\left(x_{m-1}\right)\right]}^{-1}\nabla q_{m}\left(x_{m-1}\right). \end{array}$$

With this lemma, we reformulate the Kalman filter problem into a least-square minimization problem that can be carried out sequentially. We denote

(23) $$\begin{array}{c} Z_{k}={\left(x_{0},\cdots ,x_{k}\right)}^{T}, \end{array}$$

i.e., $Z_{k}$ contains all the state variables from the initial time 0 to the current time *k*. Then, we define the objective function as follows:

(24) $$\begin{array}{c} J\left(Z_{k}\right)=\sum_{i=1}^{k}\frac{1}{2}\left[∥x_{i}-Ax_{i-1}-Bu_{i}{∥}_{{Q_{i}}^{-1}}^{2}+{∥z_{i}-Hx_{i}∥}_{{R_{i}}^{-1}}^{2}\right]+\frac{1}{2}{∥x_{0}-\mu_{0}∥}_{{Q_{0}}^{-1}}^{2} \end{array}$$

where $x_{0}$ denotes the initial state and

(25) $$\begin{array}{c} {∥x∥}_{Q^{-1}}=\sqrt{x^{T}\cdot Q^{-1}\cdot x}, \end{array}$$

with a positive definite matrix Q>0. It is assumed that the true value of $x_{0}$ follows a distribution around a certain known prior mean $\mu_{0}$ and that its uncertainty is characterized by the covariance matrix $Q_{0}$.

Based on the recursiveness of *J*, we have

(26) $$\begin{array}{c} J\left(Z_{k}\right)=J\left(Z_{k-1}\right)+\frac{1}{2}\left[∥x_{k}-Ax_{k-1}-Bu_{k}{∥}_{{Q_{k}}^{-1}}^{2}+{∥z_{k}-Hx_{k}∥}_{{R_{k}}^{-1}}^{2}\right]. \end{array}$$

Its gradient with respect to $Z_{k}$ is

(27) $$\begin{array}{c} \nabla J\left(Z_{k}\right)=\left(\begin{array}{c} \nabla J\left(Z_{k-1}\right)+{\left(0,\cdots ,0,A\right)}^{T}Q_{k}^{-1}\left(Ax_{k-1}-x_{k}+Bu_{k}\right)\\ -Q_{k}^{-1}\left(Ax_{k-1}-x_{k}+Bu_{k}\right)+H^{T}R_{k}^{-1}\left(Hx_{k}-z_{k}\right) \end{array}\right) \end{array}$$

and its Hessian is

(28) $$\nabla^{2}J\left(Z_{k}\right)\;=\;\left(\begin{array}{cc} \nabla^{2}J\left(Z_{k-1}\right)+{\left(0,\cdots ,0,A\right)}^{T}Q_{k}^{-1}\left(0,\cdots ,0,A\right) & -{\left(0,\cdots ,0,A\right)}^{T}Q_{k}^{-1}\\ -Q_{k}^{-1}\left(0,\cdots ,0,A\right) & Q_{k}^{-1}+H^{T}R_{k}^{-1}H \end{array}\right).$$

The least-square solution of the quadratic function is given by the one-step Newton’s formula

(29) $$\begin{array}{c} Z_{k}=Z_{k}^{*}-{\left[\nabla^{2}J\left(Z_{k}^{*}\right)\right]}^{-1}\nabla J\left(Z_{k}^{*}\right), \end{array}$$

where $Z_{k}^{*}$ is arbitrary.

Specifically, we choose

(30) $$\begin{array}{c} Z_{k}^{*}=\left(\begin{array}{c} {\hat{Z}}_{k-1}\\ {\hat{x}}_{k}^{*} \end{array}\right), \end{array}$$

where ${\hat{x}}_{k}^{*}$ is a prior satisfying

(31) $$\begin{array}{c} {\hat{x}}_{k}^{*}-A{\hat{x}}_{k-1}-Bu_{k}=0 \end{array}$$

and

(32) $$\begin{array}{c} \nabla J({\hat{Z}}_{k-1})=0. \end{array}$$

With this choice, the gradient reduces to

(33) $$\begin{array}{c} \nabla J\left(Z_{k}^{*}\right)=\left(\begin{array}{c} 0\\ H^{T}R_{k}^{-1}\left(H{\hat{x}}_{k}^{*}-z_{k}\right) \end{array}\right) \end{array}$$

and the Hessian is given by

(34) $$\nabla^{2}J\left({\hat{Z}}_{k}^{*}\right)\;=\;\left(\begin{array}{cc} \nabla^{2}J\left({\hat{Z}}_{k-1}\right)+{\left(0,\cdots ,0,A\right)}^{T}Q_{k}^{-1}\left(0,\cdots ,0,A\right) & -{\left(0,\cdots ,0,A\right)}^{T}Q_{k}^{-1}\\ -Q_{k}^{-1}\left(0,\cdots ,0,A\right) & Q_{k}^{-1}+H^{T}R_{k}^{-1}H \end{array}\right).$$

We then apply the one-step Newton update

$$Z_{k}=Z_{k}^{*}-{\left(\nabla^{2}J\left(Z_{k}^{*}\right)\right)}^{-1}\nabla J\left(Z_{k}^{*}\right),$$

yielding the block system

(35) $$\left[\begin{array}{cc} \nabla^{2}J\left({\hat{Z}}_{k-1}\right)+A_{\mathrm{off}}^{T}Q_{k}^{-1}A_{\mathrm{off}} & -A_{\mathrm{off}}^{T}Q_{k}^{-1}\\ -Q_{k}^{-1}A_{\mathrm{off}} & Q_{k}^{-1}+H^{T}R_{k}^{-1}H \end{array}\right]\left[\begin{array}{c} \Delta Z_{\mathrm{hist}}\\ \Delta x_{k} \end{array}\right]=-\left[\begin{array}{c} 0\\ H^{T}R_{k}^{-1}\left(Hx_{k}^{*}-z_{k}\right) \end{array}\right],$$

where $A_{\mathrm{off}}=\left(0,\ldots ,0,A\right)$ denotes the off-diagonal block of the state transition.

From the first block row of this system, we have

(36) $$\left(\nabla^{2}J\left({\hat{Z}}_{k-1}\right)+A_{\mathrm{off}}^{T}Q_{k}^{-1}A_{\mathrm{off}}\right)\Delta Z_{\mathrm{hist}}-A_{\mathrm{off}}^{T}Q_{k}^{-1}\Delta x_{k}=0.$$

Solving for $\Delta Z_{\mathrm{hist}}$ gives

(37) $$\Delta Z_{\mathrm{hist}}={\left[\nabla^{2}J\left({\hat{Z}}_{k-1}\right)+A_{\mathrm{off}}^{T}Q_{k}^{-1}A_{\mathrm{off}}\right]}^{-1}A_{\mathrm{off}}^{T}Q_{k}^{-1}\Delta x_{k}.$$

From the second block row of (35), we have

(38) $$-Q_{k}^{-1}A_{\mathrm{off}}\Delta Z_{\mathrm{hist}}+\left(Q_{k}^{-1}+H^{T}R_{k}^{-1}H\right)\Delta x_{k}=-H^{T}R_{k}^{-1}\left(Hx_{k}^{*}-z_{k}\right).$$

By eliminating $\Delta Z_{\mathrm{hist}}$ via the Schur complement, the effective Hessian for $\Delta x_{k}$ becomes

(39) $$\begin{array}{cc} S_{k} & =\left(Q_{k}^{-1}+H^{T}R_{k}^{-1}H\right)-Q_{k}^{-1}A_{\mathrm{off}}{\left[\nabla^{2}J\left({\hat{Z}}_{k-1}\right)+A_{\mathrm{off}}^{T}Q_{k}^{-1}A_{\mathrm{off}}\right]}^{-1}A_{\mathrm{off}}^{T}Q_{k}^{-1}\\ \; & =H^{T}R_{k}^{-1}H+{\left(Q_{k}+A_{\mathrm{off}}{\left(\nabla^{2}J\left({\hat{Z}}_{k-1}\right)\right)}^{-1}A_{\mathrm{off}}^{T}\right)}^{-1}. \end{array}$$

Note that $A_{\mathrm{off}}{\left(\nabla^{2}J_{k-1}\right)}^{-1}A_{\mathrm{off}}^{T}$ corresponds precisely to the predicted prior error covariance of the previous time step. Adopting the standard Kalman filter notation, this term is identified as $AP_{k-1}A^{T}$. Consequently, the composite term $\left(Q_{k}+AP_{k-1}A^{T}\right)$ is denoted as the predicted covariance $P_{k}^{-}$. Thus, the Schur complement $S_{k}$ simplifies to

(40) $$S_{k}={\left(P_{k}^{-}\right)}^{-1}+H^{T}R_{k}^{-1}H,$$

where $P_{k}^{-}$ denotes the predicted error covariance. Consequently, the Newton update $\Delta x_{k}$ is given by

(41) $$\Delta x_{k}=S_{k}^{-1}H^{T}R_{k}^{-1}\left(z_{k}-Hx_{k}^{*}\right)={\left[{\left(P_{k}^{-}\right)}^{-1}+H^{T}R_{k}^{-1}H\right]}^{-1}H^{T}R_{k}^{-1}\left(z_{k}-Hx_{k}^{*}\right).$$

Applying the SMW identity again to the gain term, we obtain the standard Kalman gain form

(42) $$K_{k}={\left[{\left(P_{k}^{-}\right)}^{-1}+H^{T}R_{k}^{-1}H\right]}^{-1}H^{T}R_{k}^{-1}=P_{k}^{-}H^{T}{\left(HP_{k}^{-}H^{T}+R_{k}\right)}^{-1}.$$

Therefore, the update equation becomes

(43) $$\Delta x_{k}=K_{k}\left(z_{k}-Hx_{k}^{*}\right),$$

which is exactly the Kalman filter measurement update. Since the cost function $J(Z_{k})$ is quadratic, the Newton step converges to the global minimum in a single iteration, making the derivation exact. The historical states $Z_{\mathrm{hist}}$ do not require updates because the chosen initial point ${\hat{Z}}_{k-1}$ already satisfies the optimality condition for the past subsystem, i.e., $\nabla J({\hat{Z}}_{k-1})=0$.

However, substituting this back into the full Newton step or recognizing that the objective function $J(Z_{k})$ is quadratic, we observe that the update for the historical states $\Delta Z_{\mathrm{hist}}$ does not need to be explicitly calculated for the recursion. The key insight is that the current state update $\Delta x_{k}$ can be computed independently by considering the Schur complement of the Hessian, leading to the standard Kalman gain formulation.

Specifically, while a non-zero innovation $\left(z_{k}-H{\hat{x}}_{k}^{*}\right)$ generally induces a correction term $\Delta Z_{\mathrm{hist}}$, which is a process characteristic of Kalman smoothers that refines historical trajectories, the standard Kalman filter operates under the Markov assumption; this assumption implies that the estimate ${\hat{Z}}_{k-1}$ is fixed for real-time recursion, effectively imposing the constraint $\Delta Z_{\mathrm{hist}}=0$.

This constraint does not imply that historical estimates are error-free; rather, it signifies that their uncertainty, characterized by the covariance $P_{k-1}$, is propagated to the current step via the system dynamics matrix A instead of being retroactively optimized. Consequently, the optimization problem is reduced to minimizing a cost function $J_{k}\left(x_{k}\right)$ solely with respect to the current state $x_{k}$, treating the predicted state ${\hat{x}}_{k}^{-}=A{\hat{x}}_{k-1}+Bu_{k}$ as a prior measurement with covariance $P_{k}^{-}=AP_{k-1}A^{T}+Q_{k}$. The resulting cost function is given by

(44) $$J_{k}\left(x_{k}\right)=\frac{1}{2}∥x_{k}-{\hat{x}}_{k}^{-}{∥}_{{\left(P_{k}^{-}\right)}^{-1}}^{2}+\frac{1}{2}{∥Hx_{k}-z_{k}∥}_{R_{k}^{-1}}^{2}.$$

Note that the weight of the prediction term is determined by the inverse of the predicted covariance ${\left(P_{k}^{-}\right)}^{-1}$ rather than the process noise covariance $Q_{k}^{-1}$, as the former accounts for the accumulated uncertainty from previous steps. Calculating the gradient and Hessian of this reduced cost function yields

(45) $$\begin{array}{cc} \nabla J_{k}\left(x_{k}\right) & ={\left(P_{k}^{-}\right)}^{-1}\left(x_{k}-{\hat{x}}_{k}^{-}\right)+H^{T}R_{k}^{-1}\left(Hx_{k}-z_{k}\right), \end{array}$$

(46) $$\begin{array}{cc} \nabla^{2}J_{k}\left(x_{k}\right) & ={\left(P_{k}^{-}\right)}^{-1}+H^{T}R_{k}^{-1}H=S_{k}. \end{array}$$

By initializing the guess at the predicted state $x_{k}^{*}={\hat{x}}_{k}^{-}$, the Newton step becomes

(47) $$\Delta x_{k}=-{\left[\nabla^{2}J_{k}\left(x_{k}\right)\right]}^{-1}\nabla J_{k}\left(x_{k}^{*}\right)=S_{k}^{-1}H^{T}R_{k}^{-1}\left(z_{k}-H{\hat{x}}_{k}^{-}\right),$$

which is mathematically identical to the standard Kalman filter update derived in (43).

**Proposition** **1.** *The Kalman filter for a linear system,* (1) *is given by the solution to the following equivalent least-squares minimization problems:*

*1.*  (48) $$\min_{Z_{k}}J\left(Z_{k}\right),$$
  *where $J(Z_{k})$ is given in* (24)*;*
*2.*  (49) $$\min_{x_{k}}J_{k}\left(x_{k}\right),$$
  *where $J_{k}\left(Z_{k}\right)$ is given in* (44)*.*

### 2.2. Extended Kalman Filters for Discrete Nonlinear Systems

The extended Kalman filter (EKF) is an extension of the Kalman filter (KF) to nonlinear systems. When system state equations or observation equations are nonlinear, the linear Kalman filter alluded to above is no longer applicable. Thus, one proposed EKF approximates the nonlinear system as a linear system through a local linearization approach, thereby retaining the recursive framework of the Kalman filter and achieving optimal estimation (minimum mean squared error) of the state of nonlinear dynamic systems [37].

We again use a discrete nonlinear system as an example. In the discrete nonlinear system, the state and measurement equations are given as follows:

(50) $$\begin{array}{c} \begin{array}{c} \begin{array}{cc} x_{k} & =g(x_{k-1},u_{k},w_{k}),\\ z_{k} & =h(x_{k},v_{k}), \end{array} \end{array} \end{array}$$

where $x_{k}\in R^{n\times 1}$ is the true state vector at step *k*, $u_{k}\in R^{m\times 1}$ is the known control input vector, $w_{k}\in R^{q\times 1}$ is the process noise, assumed to be zero-mean Gaussian with covariance $Q_{k}\in R^{q\times q}$, i.e., $w_{k}∼N\left(0,Q_{k}\right)$, $z_{k}\in R^{p\times 1}$ is the observed measurement vector, $v_{k}\in R^{r\times 1}$ is the measurement noise, assumed to be zero-mean Gaussian with covariance $R_{k}\in R^{r\times r}$, i.e., $v_{k}∼N\left(0,R_{k}\right)$, $g:R^{n}\times R^{m}\times R^{q}\to R^{n}$ is the nonlinear state transition function, and $h:R^{n}\times R^{r}\to R^{p}$ is the nonlinear observation function.

To apply the EKF, the nonlinear system is linearized using first-order Taylor expansion around the current state estimate ${\hat{x}}_{k-1}$. Let

(51) $$\begin{array}{c} {\tilde{x}}_{k}=g\left({\hat{x}}_{k-1},u_{k},0\right),\;\tilde{z}=h\left({\tilde{x}}_{k},0\right). \end{array}$$

We note that the nonlinear control terms in the discrete system does not impact the Kalman filter, so we do not need to linearize the nonlinear functions with respect to control variables.

Then, the linearized system has the following form:

(52) $$\begin{array}{cc} x_{k} & ={\tilde{x}}_{k}+A_{k}\left(x_{k}-{\hat{x}}_{k-1}\right)+W_{k}w_{k},\\ z_{k} & ={\tilde{z}}_{k}+H_{k}\left(x_{k}-{\tilde{x}}_{k}\right)+V_{k}v_{k}, \end{array}$$

where the Jacobian of *g* w.r.t. x is $A_{k}={\left.\frac{\partial g}{\partial x}\right|}_{\left({\hat{x}}_{k-1},u_{k},0\right)}\in R^{n\times n}$, the Jacobian of *g* w.r.t. w is $W_{k}={\left.\frac{\partial g}{\partial w}\right|}_{\left({\hat{x}}_{k-1},u_{k},0\right)}\in R^{n\times q}$, the Jacobian of *h* w.r.t. x is $H_{k}={\left.\frac{\partial h}{\partial x}\right|}_{\left({\tilde{x}}_{k},0\right)}\in R^{p\times n}$, and the Jacobian of *h* w.r.t. v is $V_{k}={\left.\frac{\partial h}{\partial v}\right|}_{\left({\tilde{x}}_{k},0\right)}\in R^{p\times r}$.

The noise terms in the linearized system have distributions $W_{k}w_{k}∼N\left(0,W_{k}Q_{k}W_{k}^{T}\right)$, $V_{k}v_{k}∼N\left(0,V_{k}R_{k}V_{k}^{T}\right)$.

The EKF algorithm that proceeds iteratively in two steps is formulated as follows (see Algorithm 2).

**Algorithm** **2** (Extended Kalman Filter)**.**

*(1)*  *Prediction Step.*

*Compute the a priori state estimate and error covariance:*

(53) $$\begin{array}{cc} a\;\mathrm{priori}\;\mathrm{state}\;\mathrm{estimate}:\; & {\hat{x}}_{k}^{-}=g\left({\hat{x}}_{k-1},u_{k},0\right),\\ a\;\mathrm{priori}\;\mathrm{error}\;\mathrm{covariance}:\; & P_{k}^{-}=A_{k}P_{k-1}A_{k}^{T}+W_{k}Q_{k}W_{k}^{T}, \end{array}$$

*where ${\hat{x}}_{k}^{-}\in R^{n\times 1}$ is the predicted state before incorporating new measurement and $P_{k}^{-}\in R^{n\times n}$ is the predicted error covariance matrix.*

*(2)*  *Correction Step.*

*Update the estimate using the latest measurement $z_{k}$:*

(54) $$\begin{array}{cc} \mathrm{Kalman}\;\mathrm{gain}:\; & K_{k}=P_{k}^{-}H_{k}^{T}{\left(H_{k}P_{k}^{-}H_{k}^{T}+V_{k}R_{k}V_{k}^{T}\right)}^{-1},\\ a\;\mathrm{posteriori}\;\mathrm{state}\;\mathrm{estimate}:\; & {\hat{x}}_{k}={\hat{x}}_{k}^{-}+K_{k}\left(z_{k}-h\left({\hat{x}}_{k}^{-},0\right)\right),\\ a\;\mathrm{posteriori}\;\mathrm{error}\;\mathrm{covariance}:\; & P_{k}=\left(I_{n}-K_{k}H_{k}\right)P_{k}^{-}, \end{array}$$

*where $K_{k}\in R^{n\times p}$ is the Kalman gain matrix, $I_{n}$ is the identity matrix of size n×n, and $h({\hat{x}}_{k}^{-},0)$ is the predicted measurement based on the a priori state estimate.*

For this, we cannot establish that it is an unbiased estimator for the state.

## 3. Successive Linear Estimator for Steady-State Saturated Flow

We first review the SLE method as proposed by [21] (Section 3.1, Section 3.2 and Section 3.3). Our new results begin in Section 3.4, where we reformulate SLE as an EKF and establish their mathematical equivalence. We consider the steady-state Richards equation in a spatially heterogenous porous media

(55) $$\begin{array}{c} \nabla \cdot (K(x)\nabla \phi (x))=S(x). \end{array}$$

This equation describes steady-state saturated flow, i.e., groundwater flow under dynamic equilibrium conditions, such as aquifer flow under long-term stable recharge or discharge; ϕ(x) is the hydraulic head and S(x) is the source. In the saturated flow case, the hydraulic conductivity K(x)≥0 depends only on the properties of the porous medium, and is independent of the water content; the unsaturated case, where *K* depends on the water content, is discussed in Section 4. In isotropic media, K(x) is a scalar field, while in anisotropic media it is a second-order symmetric tensor. The present study focuses on the isotropic case unless otherwise stated. In the scalar (isotropic) conductivity case, each grid node has one unknown, so the total number of independent parameters is *N*, where *N* is the number of computational grid nodes. In this anisotropic case, the tensor has six independent components at each node, leading to 6N unknowns; in a two-dimensional cross-section, this reduces to 3N unknowns. Consequently, determining the full tensor field generally requires a proportionally larger number of observations and stronger prior information. A systematic quantification of the required observation density is problem-dependent and is not pursued here. Our objective is to approximate K(x) using limited measurements of ϕ(x) and K(x) spatially.

To ensure that K(x) is non-negative, we use

(56) $$\begin{array}{c} \ln K(x)=F(x)+f(x),\;\phi =H+h(x,t), \end{array}$$

where the perturbation *f* is independent of time, F(x)=E[lnK] is the expected value of lnK when *K* is viewed as a random variable, and H=E[ϕ] is the expected value of the hydraulic head at (x,t). Both *F* and ϕ are regarded as random variables; consequently, ϕ becomes a random solution depending on *K*, and as a result *f* and *h* are random variables with zero means E[f]=E[h]=0.

Given a finite number of measurements of K(x) and ϕ(x), it may be possible to determine F(x). Before we formulate the problem rigorously, let us first review the Kriging and co-Kriging methods for obtaining linear estimators.

### 3.1. Kriging

Kriging is an elegant geostatistical method used for the optimal unbiased estimation of unknown points based on their spatial correlations [45]. Assuming that y(x) are measured at $x_{i},i=1,\cdots ,n$, where *n* is the number of the observation points, we would like to estimate y(z) at another point z via a function, as follows:

(57) $$\begin{array}{c} y\left(z\right)=F(y\left(x_{1}\right),\cdots ,y\left(x_{n}\right)) \end{array}$$

where *F* belongs to a class of functions such as linear, quadratic, or a more complicated form. If *F* is linear, then it is called a linear estimator, which is also known as Kriging. In this case, we posit the approximation as follows:

(58) $$\begin{array}{c} y_{e}\left(z\right)=\sum_{i=1}^{n}\lambda_{i}y\left(x_{i}\right) \end{array}$$

where $y_{e}\left(z\right)$ denotes the estimated value at location z and $\lambda_{i}$ are parameters to be estimated.

Unbiasedness requires that the expected estimated value be equal to the expected true value

(59) $$\begin{array}{c} \begin{array}{c} E\left[y_{e}\left(z\right)\right]=E\left[y\left(z\right)\right], \end{array} \end{array}$$

which implies

$$\sum_{i=1}^{n}\lambda_{i}=1,\;\mathrm{if}\;E\left[y\left(z\right)\right]\neq 0.$$

We hope to find a parameter $\Lambda =(\lambda_{i})$ to minimize the variance of the estimation error

(60) $$\begin{array}{c} \begin{array}{c} \min_{\Lambda }\sigma_{e}^{2}\left(z\right)=\min_{\Lambda }E\left[{\left(y\left(z\right)-y_{e}\left(z\right)\right)}^{2}\right]\\ =\min_{\Lambda }\left[C\left(z,z\right)+\sum_{i=1}^{n}\sum_{j=1}^{n}\lambda_{i}\lambda_{j}C\left(x_{i},x_{j}\right)-2\sum_{i=1}^{n}\lambda_{i}C\left(z,x_{i}\right)\right], \end{array} \end{array}$$

where $C\left(x_{i},x_{j}\right)=\mathrm{Cov}\left(y\left(x_{i}\right),y\left(x_{j}\right)\right)$ is the covariance of $y(x_{i})$ and $y(x_{j})$. Notice that $y_{e}\left(z\right)$ is a weight sum of the observed data, while $y_{e}\left(z\right)$ is the measured or estimated mean of y(z).

To estimate Λ, we introduce Lagrange multipliers η and construct the Lagrange function as follows:

(61) $$\begin{array}{c} \begin{array}{c} L\left(\Lambda ,\eta \right)=C\left(z,z\right)+\sum_{i=1}^{n}\sum_{j=1}^{n}\lambda_{i}\lambda_{j}C\left(x_{i},x_{j}\right)-2\sum_{i=1}^{n}\lambda_{i}C\left(z,x_{i}\right)-2\eta \left(1-\sum_{i=1}^{n}\lambda_{i}\right). \end{array} \end{array}$$

Utilizing the standard procedures for extremum seeking, the extremum conditions are derived as follows:

(62) $$\begin{array}{c} \begin{array}{c} \sum_{j=1}^{n}\lambda_{j}C\left(x_{k},x_{j}\right)=C\left(z,x_{k}\right)-\eta ,\;\forall k=1,\cdots ,n,\;1-\sum_{i=1}^{n}\lambda_{i}=0. \end{array} \end{array}$$

Substituting (62) into (61), we obtain the value of the Lagrange function at the extremum:

(63) $$\begin{array}{c} \begin{array}{c} L\left(\Lambda ,\eta \right)=C\left(z,z\right)-c^{T}\Lambda -\eta , \end{array} \end{array}$$

where c is an n×1 vector with elements $c_{i}=C\left(z,x_{i}\right).$ We denote C as an n×n covariance matrix with elements $C_{ij}=C\left(x_{i},x_{j}\right),$ $\Lambda ={\left[\lambda_{1},\ldots ,\lambda_{n}\right]}^{T}$ and $1={\left[1,\ldots ,1\right]}^{T}.$

The complete system is given by

(64) $$\begin{array}{c} \begin{array}{c} A\Lambda_{1}=c_{1}, \end{array} \end{array}$$

where

$$A=\left[\begin{array}{cc} C & 1\\ 1^{T} & 0 \end{array}\right],\;\Lambda_{1}=\left[\begin{array}{c} \Lambda \\ \eta \end{array}\right],\;c_{1}=\left[\begin{array}{c} c\\ 1 \end{array}\right].$$

The least-square solution is

(65) $$\begin{array}{c} \begin{array}{c} \Lambda_{1}={\left(A^{T}A\right)}^{-1}A^{T}c_{1}. \end{array} \end{array}$$

We note that the covariance c needs to be known or estimated in order to get the solution, i.e., in order to have a linear approximation using the observed data, the covariance between the estimate function value and the observed values must be known a priori.

### 3.2. Co-Kriging

Co-Kriging constructs a linear unbiased optimal estimator by jointly utilizing the spatial correlation between the target variable (primary variable) and auxiliary variables [17]. For a more accurate estimation, we use both measured values, $y\left(x_{i}\right),i=1,\cdots ,n_{y}$ (primary variable) and $h\left(x_{j}\right),j=1,\cdots ,n_{h}$ (auxiliary variable), to estimate y(z) linearly, as follows:

(66) $$\begin{array}{c} y_{e}\left(z\right)=\sum_{i=1}^{n_{y}}\lambda_{i}y\left(x_{i}\right)+\sum_{j=1}^{n_{h}}\beta_{j}h\left(x_{j}\right). \end{array}$$

The least-square solution is given by

(67) $$\begin{array}{c} \left(\Lambda ,\beta \right)=\arg\min_{\Lambda ,\beta }E\left[{\left(y\left(z\right)-y_{e}\left(z\right)\right)}^{2}\right]. \end{array}$$

The solution is governed by the following linear equation system:

(68) $$\begin{array}{c} \left[\begin{array}{cc} C_{yy} & C_{yh}\\ C_{hy} & C_{hh} \end{array}\right]\left[\begin{array}{c} \lambda \\ \beta \end{array}\right]=\left[\begin{array}{c} c_{y}\\ c_{h} \end{array}\right] \end{array}$$

where the covariance matrices are defined by

(69) $$\begin{array}{c} \begin{array}{c} C_{yh}\left(x_{i},x_{j}\right)=\mathrm{Cov}\left[y\left(x_{i}\right),h\left(x_{j}\right)\right]. \end{array} \end{array}$$

The covariance matrices with subscripts yy,hy,hh are defined analogously. The covariance vectors on the right hand side are defined by

(70) $$\begin{array}{c} \begin{array}{c} {\left(c_{y}\right)}_{i}=\mathrm{Cov}\left[y\left(z\right),y\left(x_{i}\right)\right],i=1,\cdots ,n_{y},\\ {\left(c_{h}\right)}_{j}=\mathrm{Cov}\left[y\left(z\right),h\left(x_{j}\right)\right],j=1,\cdots ,n_{h}. \end{array} \end{array}$$

To construct an unbiased estimator, constraints can be employed as in the case of Kriging. Two constraints in co-Kriging are given by

(71) $$\begin{array}{c} \sum_{i=1}^{n_{y}}\lambda_{i}=1,\;\sum_{j=1}^{n_{h}}\beta_{j}=0. \end{array}$$

Once again, the covariances must be known or estimated to obtain the solution.

The normal equations are

(72) $$\begin{array}{c} \left[\begin{array}{cccc} C_{yy} & 1 & C_{yh} & 0\\ 1^{T} & 0 & 0^{T} & 0\\ C_{hy} & 0 & C_{hh} & 1\\ 0^{T} & 0 & 1^{T} & 0 \end{array}\right]\left[\begin{array}{c} \lambda \\ \eta_{y}\\ \beta \\ \eta_{h} \end{array}\right]=\left[\begin{array}{c} c_{y}\\ 1\\ c_{h}\\ 0 \end{array}\right], \end{array}$$

where $\eta_{y}$ and $\eta_{h}$ are two Lagrange multipliers used for enforcing unbiasedness of the linear estimator.

### 3.3. Successive Linear Estimator

In the context of SLE for hydraulic conductivity, the state equation is given by

(73) $$\ln K={\hat{Y}}_{c}+f,\;f∼N\left(0,R_{ff}\right),\;R_{ff}=E\left[ff^{T}\right],$$

where ${\hat{Y}}_{c}=E\left[\ln K\right]$ and E[f]=0. In practice, $R_{ff}$ is specified from prior geostatistical information, such as a variogram model fitted to available conductivity measurements or inferred from geological facies statistics. The Gaussian assumption is a modeling choice for the prior distribution, not a physical assertion about the medium itself. The observation equation is given implicitly by

(74) $$\begin{array}{c} \begin{array}{c} \phi =F\left(\ln K\right),\Leftrightarrow \nabla \cdot (e^{\ln K}\nabla \phi \left(x\right))-S=0, \end{array} \end{array}$$

where F is the mapping that maps lnK to the solution of the Richards equation, K(x) is the saturated hydraulic conductivity, ϕ(x) is the total hydraulic head, and

$$h=\phi -E\left[\phi \right]∼N\left(0,R_{hh}\right),\;R_{hh}=E\left[hh^{T}\right].$$

The successive linear estimator at a location $x_{o}$ for lnK is given iteratively by

(75) $$\begin{array}{c} \begin{array}{c} {\hat{Y}}_{c}^{\left(r+1\right)}\left(x_{o}\right)={\hat{Y}}_{c}^{\left(r\right)}\left(x_{o}\right)+\sum_{j=n_{f}+1}^{n_{f}+n_{h}}\omega_{j}^{\left(r\right)}\left[\phi^{*}\left(x_{j}\right)-\phi^{\left(r\right)}\left(x_{j}\right)\right], \end{array} \end{array}$$

where ${\hat{Y}}_{c}^{\left(r\right)}\left(x_{o}\right)$ is the r-th iteration of the estimated conditional mean of lnK, *r* is the iteration index, $\omega_{j}$ is the model weight parameter, $\phi^{*}$ denotes the observation, and $\phi^{\left(r\right)}$ is the outcome of the observation equation or the solution of the Richards equation (model-computed value) at the *r*-th iteration.

To ensure the estimator having minimal variance, the goal is to minimize the variance with respect to ω’s

(76) $$\begin{array}{c} \begin{array}{c} \min E_{\omega^{\left(r\right)}}\left[{\left(\ln K-{\hat{Y}}_{c}^{\left(r+1\right)}\right)}^{2}\right]. \end{array} \end{array}$$

We differentiate the squared error with respect to $\omega^{\left(r\right)}$ and set the resultant to zero to yield

(77) $$\begin{array}{c} \begin{array}{c} \sum_{j=n_{f}+1}^{n_{f}+n_{h}}\omega_{j}^{\left(r\right)}\varepsilon_{hh}^{\left(r\right)}\left(x_{j},x_{l}\right)=\varepsilon_{fh}^{\left(r\right)}\left(x_{o},x_{l}\right), \end{array} \end{array}$$

where $h=\phi^{*}-\phi ,\;f=\ln K-{\hat{Y}}_{c},\;\varepsilon_{hh}=E\left[hh\right],\;\varepsilon_{fh}=E\left[fh\right].$ In the following, we set ${\hat{Y}}_{c}=0$.

The iteration process of SLE is summarized Algorithm 3.

**Algorithm** **3** (SLE)**.**

*(1)*  *Initialization.*

*The prior estimate is obtained using the classical co-Kriging technique:*

(78) $$\begin{array}{c} \begin{array}{c} {\hat{Y}}_{c}^{\left(0\right)}\left(x_{o}\right)=\sum_{i=1}^{n_{f}}\lambda_{io}f_{i}^{*}\left(x_{i}\right)+\sum_{j=n_{f}+1}^{n_{f}+n_{h}}\mu_{jo}h_{j}^{*}\left(x_{j}\right) \end{array} \end{array}$$

*where $f_{i}^{*}\left(x_{i}\right)=\ln K^{*}\left(x_{i}\right)-{\hat{Y}}_{c}\left(x_{i}\right)$ denotes the de-meaned log-conductivity observation at the i-th measurement location and $h_{j}^{*}\left(x_{j}\right)=\phi^{*}\left(x_{j}\right)-E\left[\phi \left(x_{j}\right)\right]$ denotes the de-meaned hydraulic head observation at the j-th measurement location. The superscript ∗ indicates observed data, while λ and μ are calculated by the following equations:*

(79) $$\begin{array}{c} \begin{array}{c} \sum_{i=1}^{n_{f}}\lambda_{io}R_{ff}\left(x_{i},x_{l}\right)+\sum_{j=n_{f}+1}^{n_{f}+n_{h}}\mu_{jo}R_{hf}\left(x_{j},x_{l}\right)=R_{ff}\left(x_{o},x_{l}\right),\;l=1,2,\cdots ,n_{f},\\ \sum_{i=1}^{n_{f}}\lambda_{io}R_{fh}\left(x_{i},x_{l}\right)+\sum_{j=n_{f}+1}^{n_{f}+n_{h}}\mu_{jo}R_{hh}\left(x_{j},x_{l}\right)=R_{fh}\left(x_{o},x_{l}\right),\;l=n_{f}+1,n_{f}+2,\cdots ,n_{f}+n_{h}. \end{array} \end{array}$$

*For notational simplicity, the asterisk ∗ on the observed quantities is omitted in the covariance matrices, i.e., $R_{ff}$ and $\varepsilon_{ff}$ are understood to be constructed from the observed $f^{*}$ and $h^{*}$.*

*The prior covariance is*

(80) $$\begin{array}{c} \begin{array}{c} \varepsilon_{ff}^{\left(1\right)}\left(x_{o},x_{k}\right)=R_{ff}\left(x_{o},x_{k}\right)-\sum_{i=1}^{n_{f}}\lambda_{io}R_{ff}\left(x_{o},x_{i}\right)-\sum_{j=n_{f}+1}^{n_{f}+n_{h}}\mu_{jo}R_{fh}\left(x_{k},x_{j}\right). \end{array} \end{array}$$

*The covariance matrices $R_{ff}$, $R_{fh}$, and $R_{hh}$ are constructed from prior spatial statistics (e.g., exponential or Gaussian variogram models) with parameters estimated from available data or taken from the literature for similar hydrogeological settings.*

*(2)*  *Prediction (r + 1)-th step.*

*At the current (r + 1)-th step, the optimal solution ${\hat{Y}}_{c}^{\left(r\right)}$ from the previous step (i.e., the r-th step) is treated as the state prior for this step. Meanwhile, the error covariance $\varepsilon_{ff}^{\left(r\right)}$ from the previous step is taken as the error covariance prior for this step.*

*(3)*  *Correction (r + 1)-th step.*

*Similar to the Kalman gain, $\omega^{\left(r\right)}$ is given by*

(81) $$\begin{array}{c} \begin{array}{c} \sum_{j=n_{f}+1}^{n_{f}+n_{h}}\omega_{j}^{\left(r\right)}\varepsilon_{hh}^{\left(r\right)}\left(x_{j},x_{l}\right)=\varepsilon_{fh}^{\left(r\right)}\left(x_{o},x_{l}\right),\;l=n_{f}+1,\cdots ,n_{f}+n_{h}, \end{array} \end{array}$$

*where*

(82) $$\begin{array}{cc} \varepsilon_{hh}^{\left(r\right)} & =J^{\left(r\right)}\varepsilon_{ff}^{\left(r\right)}J^{{\left(r\right)}^{T}},\\ \varepsilon_{fh}^{\left(r\right)} & =J^{\left(r\right)}\varepsilon_{ff}^{\left(r\right)}, \end{array}$$

*and the Jacobian matrix (Sensitivity matrix) J of the observation equation is given by*

(83) $$\begin{array}{c} \begin{array}{c} J^{\left(r\right)}={\left.\frac{\partial F}{\partial \ln K}\right|}_{{\hat{Y}}_{c}^{\left(r\right)}}. \end{array} \end{array}$$

*This Jacobian is evaluated by an adjoint-based sensitivity analysis of the discretized Richards equation, which yields the derivative of the simulated hydraulic head with respect to perturbations in lnK at each iteration.*

*In practical calculations, an additional relaxation term *Θ* is usually added to the diagonal of the matrix in* (81) *during each iteration:*

(84) $$\begin{array}{c} \begin{array}{c} \sum_{j=n_{f}+1}^{n_{f}+n_{h}}\omega_{j}^{\left(r\right)}\varepsilon_{hh}^{\left(r\right)}\left(x_{j},x_{l}\right)+\Theta^{\left(r\right)}\delta_{jl}=\varepsilon_{fh}^{\left(r\right)}\left(x_{o},x_{l}\right),\;\;l=n_{f}+1,\cdots ,n_{f}+n_{h}. \end{array} \end{array}$$

*This is actually an $L_{2}$ regularization (also known as the Tikhonov regularization or ridge regression). From a Bayesian perspective, it is equivalent to the parameter $\omega_{j}^{\left(r\right)}$ following a Gaussian distribution with mean 0 and variance proportional to 1/Θ. Functionally, *Θ* can be interpreted as a virtual observation noise, which makes the system perceive the observed data as noisier than it actually is, thereby suppressing overfitting and numerical oscillations.*

*The posterior estimate is then given by*

(85) $$\begin{array}{c} \begin{array}{c} {\hat{Y}}_{c}^{\left(r+1\right)}\left(x_{o}\right)={\hat{Y}}_{c}^{\left(r\right)}\left(x_{o}\right)+\sum_{j=n_{f}+1}^{n_{f}+n_{h}}\omega_{j}^{\left(r\right)}\left[\phi_{j}^{*}\left(x_{j}\right)-\phi_{j}^{\left(r\right)}\left(x_{j}\right)\right], \end{array} \end{array}$$

*where $\phi_{j}^{*}\left(x_{j}\right)$ are given. The error covariance is updated as follows:*

(86) $$\begin{array}{c} \begin{array}{c} \varepsilon_{ff}^{\left(r+1\right)}\left(x_{o},x_{k}\right)=\varepsilon_{ff}^{\left(r\right)}\left(x_{o},x_{k}\right)-\sum_{l=n_{f}+1}^{n_{f}+n_{h}}\omega_{l}^{\left(r\right)}\varepsilon_{fh}^{\left(r\right)}\left(x_{k},x_{l}\right). \end{array} \end{array}$$

### 3.4. Reformulation of the SLE as an EKF

This subsection contains one of the main contributions of this paper. We show that the successive linear estimator (SLE) is essentially a specific application of the extended Kalman filter (EKF) in static parameter estimation using the Richards equation. The equivalence stated below holds under three key assumptions: (i) the state is static with identity dynamics and zero process noise; (ii) the nonlinear observation operator is linearized by first-order Taylor expansion at each iteration; and (iii) the prior is Gaussian and the initial state and covariance are supplied by co-Kriging. Under these conditions, the iterative SLE update is mathematically identical to the EKF prediction–correction scheme, as we demonstrate below.

We distinguish three levels of equivalence in this paper: Section 2 establishes the exact linear-Gaussian equivalence between the Kalman filter and weighted least squares; by contrast, the EKF update is a Gauss–Newton type approximation due to first-order linearization; finally, the SLE–EKF correspondence presented here builds upon this latter level and shows that the iterative SLE update is identical to the iterative EKF update under the assumptions above.

In the problem of estimating the hydraulic conductivity, the logarithmic conductivity lnK(x) is defined as the state variable and the hydraulic head ϕ(x) is defined as the observation variable. A nonlinear model system is established based on the solution solver of the Richards equation with the given hydraulic conductivity lnK(x).

This is a static parameter estimation problem, so the state transition function is an identity mapping augmented with a Gaussian noise

(87) $$x_{k}=x_{k-1}+w_{k},\;\left(x\leftarrow {\hat{Y}}_{c}\right).$$

Within the SLE framework, the process noise $w_{k}\equiv 0$, i.e., $Q=E[w_{k}w_{k}^{T}]=0$. The condition of Q=0 indicates a state of complete confidence that the state (i.e., lnK) will not undergo unpredictable changes during the iteration process. Its uncertainty propagates solely through the initial covariance matrix $P_{0}^{-}$ and the Jacobian matrix J presented below. Here, $x_{k}={\hat{Y}}_{c}^{\left(k\right)}\in R^{N\times 1}$ is the conditional mean of lnK(x) at the *k*-th iteration (the state to be estimated).

The observation equation with a Gaussian noise is given by

(88) $$z_{k}=F\left(x_{k}\right)+v_{k},\;\left(z\leftarrow \phi \right),$$

where $z_{k}\in R^{n_{h}\times 1}$ and F is the observation operator, i.e., $z_{k}$ is the solution of the steady-state Richards equation $\nabla \cdot \left(e^{x_{k}}\nabla z_{k}\right)=S\left(x\right)$. Without loss of generality, we assume S(x)=0 in the following. The measurements are assumed error-free, with the observation noise $v_{k}\equiv 0$. As a result, the covariance of the observation noise $R_{k}=E\left[v_{k}v_{k}^{T}\right]=0$. We emphasize that this is a theoretical simplification for the SLE–EKF equivalence derivation. In actual computations, the regularization parameter Θ, introduced in the SLE algorithm, plays the equivalent role of a nonzero observation noise covariance $R_{k}$, ensuring the stability of the inversion in the presence of measurement errors. Thus, the practical implementation is not noise-free; rather, the uncertainty is represented through Θ instead of $R_{k}$.

One defines the state error as $e_{k}=x_{k}-{\hat{x}}_{k}=\ln K-{\hat{Y}}_{c}$. The objective of the error estimation is to minimize the covariance of the state error with respect to parameter $K_{k}$, i.e.,

(89) $$\begin{array}{c} \min_{K_{k}}\mathrm{tr}\left(E\left[e_{k}e_{k}^{T}\right]\right). \end{array}$$

Denote ${\hat{Y}}_{c}$ as the unbiased estimator of lnK, that is, $E[{\hat{Y}}_{c}]=\ln K$; it follows that $E[e_{k}]=0$.

**Initialization.**

The prior state ${\hat{x}}_{0}^{-}={\hat{Y}}_{c}^{\left(0\right)}\in R^{N\times 1}$ is determined by the co-Kriging method using both the conductivity and hydraulic head observations through the following equation system:

(90) $${\hat{x}}_{0}^{-}=\Lambda f^{\prime }+Mh^{\prime }$$

where $f^{\prime }\in R^{n_{f}\times 1}$ is the de-meaned logarithmic hydraulic conductivity vector with $f_{i}^{\prime }=\ln K\left(x_{i}\right)-E\left[\ln K\right]$ ($i=1,\cdots ,n_{f}$), $h^{\prime }\in R^{n_{h}\times 1}$ is the de-meaned hydraulic head vector with $h_{j}^{\prime }=\phi \left(x_{j}\right)-E\left[\phi \right]$ ($j=1,\cdots ,n_{h}$), $\Lambda \in R^{N\times n_{f}}$ is the global Kriging weight matrix for hydraulic conductivity observations with ${\left[\Lambda \right]}_{o,i}=\lambda_{io}$ (o=1,⋯,N; $i=1,\cdots ,n_{f}$), $M\in R^{N\times n_{h}}$ is the global Kriging weight matrix for hydraulic head observations with ${\left[M\right]}_{o,j}=\mu_{jo}$ (o=1,⋯,N; $j=1,\cdots ,n_{h}$), *N* is the total number of spatial nodes to be estimated, $n_{f}$ is the number of hydraulic conductivity observation points, and $n_{h}$ is the number of hydraulic head observation points.

Here, Λ and *M* are determined by the following system of linear equations:

(91) $$\left[\begin{array}{cc} R_{ff,obs} & R_{hf,obs}\\ R_{fh,obs} & R_{hh,obs} \end{array}\right]\left[\begin{array}{c} \lambda_{o}\\ \mu_{o} \end{array}\right]=\left[\begin{array}{c} r_{ff,o}\\ r_{fh,o} \end{array}\right]$$

where $\lambda_{o}\in R^{n_{f}\times 1}$ is the weight sub-vector corresponding to the hydraulic conductivity observations for the target node $x_{o}$ (the *o*-th row of Λ) and $\mu_{o}\in R^{n_{h}\times 1}$ is the weight sub-vector corresponding to the hydraulic head observations for the target node $x_{o}$ (the *o*-th row of M), $R_{ff,obs}\in R^{n_{f}\times n_{f}}$ is the autocovariance matrix among the hydraulic conductivity observation points with ${\left[R_{ff,obs}\right]}_{i,l}=R_{ff}\left(x_{i},x_{l}\right)$ ($i,l=1,\cdots ,n_{f}$), $R_{hh,obs}\in R^{n_{h}\times n_{h}}$ is the autocovariance matrix among the hydraulic head observation points with ${\left[R_{hh,obs}\right]}_{j,l}=R_{hh}\left(x_{j},x_{l}\right)$ ($j,l=1,\cdots ,n_{h}$), $R_{fh,obs}\in R^{n_{f}\times n_{h}}$ is the cross-covariance matrix between the hydraulic conductivity and hydraulic head observation points with ${\left[R_{fh,obs}\right]}_{i,j}=R_{fh}\left(x_{i},x_{j}\right)$ ($i=1,\cdots ,n_{f}$; $j=1,\cdots ,n_{h}$) and $R_{hf,obs}=R_{fh,obs}^{T}$, $r_{ff,o}\in R^{n_{f}\times 1}$ is the autocovariance vector between the target node $x_{o}$ and the hydraulic conductivity observation points with ${\left[r_{ff,o}\right]}_{l}=R_{ff}\left(x_{o},x_{l}\right)$ ($l=1,\cdots ,n_{f}$), and $r_{fh,o}\in R^{n_{h}\times 1}$ is the cross-covariance vector between the target node $x_{o}$ and the hydraulic head observation points with ${\left[r_{fh,o}\right]}_{l}=R_{fh}\left(x_{o},x_{l}\right)$ ($l=1,\cdots ,n_{h}$).

The value of the initial prior covariance $P_{0}^{-}=\varepsilon_{ff}^{\left(0\right)}\in R^{N\times N}$ is the conditional covariance after incorporating some observations ($n_{f}$ conductivity observations and $n_{h}$ hydraulic head observations) through one-step classical co-Kriging:

(92) $$P_{0}^{-}=R_{ff}-\Lambda R_{ff,obs}\Lambda^{T}-MR_{fh}M^{T}$$

where $R_{ff}\in R^{N\times N}$ is the global autocovariance matrix of the logarithmic conductivity perturbation term *f* with ${\left[R_{ff}\right]}_{a,b}=R_{ff}\left(x_{a},x_{b}\right)$ (a,b=1,⋯,N) and $R_{fh}\in R^{N\times n_{h}}$ is the global cross-covariance matrix between the logarithmic conductivity perturbation term *f* and the hydraulic head perturbation term *h* with ${\left[R_{fh}\right]}_{a,j}=R_{fh}\left(x_{a},x_{j}\right)$ (a=1,⋯,N; $j=1,\cdots ,n_{h}$). The complete SLE algorithm reformulated in the EKF framework is given in Algorithm 4.

**Algorithm** **4** (SLE Algorithm formulated in EKF framework)**.**

*(1)*  *Prediction Step:*

*Based on the previous state estimate ${\hat{x}}_{k-1}$ (posterior estimate), predict the prior state and prior error covariance of the current iteration:*

(93) $$\begin{array}{cc} \mathrm{prior}\;\mathrm{state}\;\mathrm{estimate}:\; & {\hat{x}}_{k}^{-}={\hat{x}}_{k-1}\\ \mathrm{prior}\;\mathrm{error}\;\mathrm{covariance}:\; & P_{k}^{-}=P_{k-1}+Q_{k}\in R^{N\times N} \end{array}$$

*when $Q_{k}=0$, $P_{k}^{-}=\varepsilon_{ff}^{\left(k\right)}.$ Since the state equation has no dynamic evolution and is only a static estimate, the state estimate remains the result of the previous optimization in the absence of new observations, and the state uncertainty remains unchanged in the absence of new information. However, in practical groundwater systems the hydraulic conductivity may exhibit unmodeled minor dynamic perturbations due to factors such as medium heterogeneity, temperature variations, and pressure fluctuations; these perturbations are referred to as process noise.*

*(2)*  *Correction Step:*

*Combined with the hydraulic head observation data $z_{k}=\phi^{*}\left(x\right)$ of the current iteration, complete an iterative optimization by linearizing the observation equation, calculating the Kalman gain, and correcting the state estimate and error covariance.*

*Perform a first-order Taylor expansion of the nonlinear observation operator F(x) at the current prior state ${\hat{x}}_{k}^{-}$ (i.e., the posterior ${\hat{x}}_{k-1}$ of the previous step). The linearized observation equation is*

(94) $$z_{k}={\tilde{z}}_{k}+J_{k}\left(x_{k}-{\hat{x}}_{k-1}\right)+v_{k},$$

*where ${\tilde{z}}_{k}=F\left({\hat{x}}_{k-1}\right)$ and $J_{k}={\left.\frac{\partial F}{\partial x}\right|}_{{\hat{x}}_{k-1}}\in R^{n_{h}\times N}$ is the Jacobian matrix (sensitivity matrix) of the observation equation. The Jacobian $J_{k}$ is evaluated by the same adjoint-based sensitivity approach used for the SLE:*

(95) $$\begin{array}{cc} \mathrm{Kalman}\;\mathrm{gain}:\; & K_{k}=P_{k}^{-}J_{k}^{T}{\left(J_{k}P_{k}^{-}J_{k}^{T}+R_{k}\right)}^{-1},\\ \mathrm{posterior}\;\mathrm{state}\;\mathrm{estimate}:\; & {\hat{x}}_{k}={\hat{x}}_{k}^{-}+K_{k}\left(z_{k}-{\tilde{z}}_{k}\right),\\ \mathrm{posterior}\;\mathrm{error}\;\mathrm{covariance}:\; & P_{k}=\left(I-K_{k}J_{k}\right)P_{k}^{-}, \end{array}$$

*where $K_{k}\in R^{N\times n_{h}},{\left[K_{k}\right]}_{o,j}=\omega_{jo}^{\left(k\right)}\in R^{N\times n_{h}}$, ${\hat{x}}_{k}={\hat{Y}}_{c}^{\left(k\right)}$, ${\hat{x}}_{k}^{-}={\hat{Y}}_{c}^{\left(k-1\right)}$, $z_{k}=\phi^{*}\left(x\right)$, ${\tilde{z}}_{k}=\phi^{\left(k\right)}\left(x\right)$, $P_{k}^{-}=\varepsilon_{ff}^{\left(k-1\right)}$, and $P_{k}=\varepsilon_{ff}^{\left(k\right)}$.*

*Here, $\varepsilon_{ff}^{\left(k-1\right)}$ denotes the error covariance matrix of the log-conductivity estimate at the (k−1)-th iteration, defined as*

$$\varepsilon_{ff}^{\left(k-1\right)}=E\left[\left(\ln K-{\hat{Y}}_{c}^{\left(k-1\right)}\right){\left(\ln K-{\hat{Y}}_{c}^{\left(k-1\right)}\right)}^{T}\right],$$

*where ${\hat{Y}}_{c}^{\left(k-1\right)}$ is the conditional mean estimate from the previous iteration. This corresponds to the prior error covariance before the k-th correction step. However, in practical measurements, observation errors are inevitable due to sensor precision limitations and environmental interference (e.g., water level fluctuations). The larger the value of $R_{k}$, the less accurate the observation; correspondingly, the Kalman gain $K_{k}$ becomes smaller, leading to a greater reliance on the a priori estimate.*

From a mathematical perspective, both $R_{k}$ and the regularization parameter Θ that can be added as a $L_{2}$ regularization of the least squares optimization serve to stabilize matrix inversion; the presence of $R_{k}$ ensures that the matrix $J_{k}P_{k}^{-}J_{k}^{T}+R_{k}$ is positive definite, allowing for safe inversion and avoiding numerical instability. In other words, when solving a linear system, both Θ and $R_{k}$ play the role of regularization terms, which are used to avoid the matrix being ill-conditioned. From a Bayesian viewpoint, both $R_{k}$ and Θ represent prior information. The Θ term implicitly assumes that the Kalman gain $\omega_{jo}$ itself follows a Gaussian distribution centered at 0 with variance 1/Θ; this is a type of prior distribution on the parameters. The $R_{k}$ term represents the assumption that the observation $z_{k}$ follows a Gaussian distribution centered at the true value with variance $R_{k}$; this is a type of prior distribution on the data. Although $R_{k}$ and Θ have equivalent roles, their origins are not the same; $R_{k}$ models the uncertainty in the data within the Bayesian framework, whereas Θ is an artificially introduced regularization term that models the uncertainty in the parameters.

By all means, we claim that the EKF outcome defined here is exactly the iterative solution of the SLE. This is the primary objective of the present paper.

## 4. Parameter Estimation for Transient Unsaturated Flow Using the Extended Kalman Filter

This section presents another main contribution of the paper, namely, extending the unified SLE–EKF framework established in Section 3.4 to transient unsaturated flow. The general form of the transient Richards equation for unsaturated flow is given by

(96) $$\begin{array}{c} \begin{array}{c} \frac{\partial \theta (\phi (x,t))}{\partial t}+S\left(x,t\right)=\nabla \cdot \left[K\left(\theta ,x\right)\nabla \left(\phi (x,t)+z\right)\right], \end{array} \end{array}$$

where θ(x,t) is the volumetric water content, ϕ as the pressure head is normally negative, *z* is the elevation head, ϕ+z is the total hydraulic head, ∇(ϕ+z) is the gradient of the total hydraulic head, which includes both the pressure gradient and the gravitational gradient, K(θ,x) is the unsaturated hydraulic conductivity, which is strongly coupled to the water content θ, θ(ϕ) is a monotonically increasing function of ϕ, and K(θ,x) is a monotonically increasing function of θ as well as a monotonically increasing function of ϕ. In unsaturated soil, lower water content reduces pore-water connectivity, resulting in a smaller *K*; at saturation, *K* reaches its maximum value $K_{s}\left(x\right)$ (the saturated hydraulic conductivity). The pressure head ϕ and water content θ are in one-to-one correspondence via the soil–water retention curve (SWRC), expressed as θ=θ(ϕ).

Given the Richards equation with conductivity K(θ,x)>0, we would like to estimate K(θ,x) through a few measurements of $K\left(\theta \left(x_{i},t_{k}\right),x_{i}\right),i=1\cdots ,n_{f}$ and $\phi \left(x_{j},t_{k}\right),j=n_{f}+1,\cdots ,n_{f}+n_{h},k=1,\cdots ,N_{t}$, subject to given initial and boundary conditions. Since K>0, one normally uses

(97) $$\begin{array}{c} \ln K(\theta ,x)=F(x)+f(\theta ,x) \end{array}$$

as the effective variable to ensure its positivity, where F=E[lnK] is the spatial mean of the log-saturated conductivity and *f* is the small fluctuation with E[f]=0 and E[(f,f)]=Q(x)>0. Analogously, we set

(98) $$\begin{array}{c} \phi (x,t)=H(x,t)+h(x,t), \end{array}$$

where H=E[ϕ] is the mean of ϕ and *h* is the perturbation, with E[h]=0 and E[h,h]=R(x,t)>0.

This inverse problem could also be formulated using the EKF. The objective here is to estimate K(θ,x) using the spatial–temporal observation data. The state equation for *K* and the observation equation for ϕ are respectively defined by

(99) $$\begin{array}{c} \begin{array}{c} K(\theta ,x)=K(\theta ,x)\;(\mathrm{static}\;\mathrm{parameter}),\;F(K)=\phi , \end{array} \end{array}$$

where the hydraulic conductivity is a static parameter that does not evolve with time; its state equation is simply an identity mapping. Moreover, F represents the time-dependent inverse operator defined by the solution solver of the Richards equation; however, exploiting the time-dependence of F separates this inverse problem from the saturated case alluded to in the previous section.

To highlight the key differences between the steady-state saturated (Section 3) and transient unsaturated (Section 4) formulations, we summarize the two frameworks in Table 1 below. This unified framework supports typical hydrogeological inverse problems, including hydraulic tomography and transient vadose zone calibration.

The comparison in Table 1 clarifies the different physical settings, governing equations, parameterization strategies, and inversion approaches used in the two sections.

### 4.1. Parametric Inversion Aided by the Van Genuchten–Mualem Model

To make the problem tractable, we assume that the hydraulic conductivity admits a separable form that separates the spatial variability of the saturated hydraulic conductivity $K_{s}\left(x\right)$ from the universal dependence of the relative hydraulic conductivity on pressure head $K_{r}\left(\theta \right)$, as follows:

(100) $$\begin{array}{c} \begin{array}{c} K\left(\theta ,x\right)=K_{s}\left(x\right)\;K_{r}\left(\theta \right) \end{array} \end{array}$$

where $K_{s}\left(x\right)$ is the maximum hydraulic conductivity and $K_{r}\left(\theta \right)$ is given by an empirical constitutive law such as the van Genuchten–Mualem model. For de-meaned $\ln K_{s}\left(x\right)$, we assume that it can be expanded in a set of orthogonal basis functions ${\left\{\varphi_{i}\left(x\right)\right\}}_{i=1}^{M}$. The choice of orthogonal system is not restricted a priori; on regular domains (e.g., rectangular or layered), classical bases such as Fourier series, cosine or sine bases (suited to specific boundary conditions), and orthogonal polynomials (e.g., Legendre, Chebyshev) are commonly employed. For problems where multi-scale or localized spatial features are anticipated, wavelet bases (e.g., Haar, Daubechies) provide alternatives with compact support in both space and scale. Of course, we could also consider the Karhunen–Loève expansion.

After the orthogonal basis is determined, we expand $\ln K_{s}\left(x\right)$ as follows:

(101) $$\begin{array}{c} \begin{array}{c} \ln K_{s}\left(x\right)=F\left(x\right)+\sum_{i=1}^{M}c_{i}\;\varphi_{i}\left(x\right),\;E\left[c_{i}\right]=0, \end{array} \end{array}$$

where $F\left(x\right)=E[\ln K_{s}\left(x\right)]$ is the prior mean field, prescribed as a constant or a spatial trend based on available information (e.g., geological interpretation or direct measurements of $\ln K_{s}$) and $c={\left[c_{1},c_{2},\ldots ,c_{M}\right]}^{T}\in R^{M}$ is the vector of unknown coefficients. The prior covariance of c is denoted by $\Sigma_{c}\in R^{M\times M}$. The prior covariance $\Sigma_{c}$ is prescribed based on the spatial correlation structure of $\ln K_{s}$, typically using a stationary covariance kernel with correlation length and variance inferred from site-specific data or regional analogs. The coefficients c are treated as random variables in order to reflect our incomplete knowledge of the spatial field; this is a Bayesian prior formulation rather than a claim of intrinsic randomness.

The expansion in (101) serves two purposes. First, it reduces the dimensionality of the estimation problem from the number of grid nodes *N* (typically $10^{3}$–$10^{5}$) to a much smaller number of coefficients *M* (typically $10^{1}$–$10^{2}$). This compression is possible because the spatial correlation of $\ln K_{s}$ implied by the prior covariance $\Sigma_{c}$ allows the random field to be accurately represented by the leading *M* basis functions. Second, the orthogonality of $\left\{\varphi_{i}\right\}$ simplifies the subsequent co-Kriging and EKF computations by providing a diagonal or nearly diagonal prior covariance structure for the coefficients. The truncation level *M* is chosen such that the retained basis functions capture a prescribed fraction (e.g., 90–95%) of the total prior variance, i.e.,

$$\frac{\sum_{i=1}^{M}\lambda_{i}}{\sum_{i=1}^{\infty }\lambda_{i}}\approx 0.9--0.95,$$

where $\lambda_{i}$ are the eigenvalues of the prior covariance operator associated with $\left\{\varphi_{i}\right\}$. In practice, *M* is determined by the decay rate of the eigenvalue spectrum and is typically much smaller than *N*, making the inversion computationally feasible.

The prior mean field F(x) is kept fixed throughout the inversion; it does not appear in the state vector and is not updated by the EKF. At each iteration, the total $\ln K_{s}$ field is reconstructed by adding F(x) to the fluctuation estimated from the current state $x_{k}$ and the Richards equation is solved using this total field to compute the predicted pressure head. The objective of the inverse problem is essentially to estimate the unknown expansion coefficients $\left\{c_{i}\right\}$. The relative hydraulic conductivity $K_{r}\left(\theta \right)$ and the soil–water retention curve θ(ϕ) are prescribed by the van Genuchten–Mualem model, detailed below. The complete unsaturated hydraulic conductivity is then given by

(102) $$\begin{array}{c} \begin{array}{c} K\left(\theta ,x\right)=K_{s}\left(x\right)\;K_{r}\left(\theta \right)=\exp\;\left(F\left(x\right)+\sum_{i=1}^{M}c_{i}\varphi_{i}\left(x\right)\right)\cdot K_{r}\left(\theta \right). \end{array} \end{array}$$

Because $K_{r}\left(\theta \right)$ is a monotonically increasing function of θ, and hence of ϕ, and because the prefactor $\exp(F\left(x\right)+\sum c_{i}\varphi_{i}\left(x\right))$ is strictly positive, the product K(θ,x) inherits the monotonicity with respect to ϕ required by physics.

The relative hydraulic conductivity $K_{r}\left(\theta \right)$ appearing in (102) is defined by the van Genuchten–Mualem model [9,10]. In this model, the effective water saturation $S_{e}$ is expressed as a function of the pressure head ϕ

(103) $$\begin{array}{c} \begin{array}{c} S_{e}\left(\phi \right)=\frac{\theta \left(\phi \right)-\theta_{r}}{\theta_{s}-\theta_{r}}=\left\{\begin{array}{cc} \frac{1}{{\left[1+(\alpha |\phi |\right)}^{n}{]}^{m}}, & \phi <0,\\ 1, & \phi \geq 0, \end{array}\right. \end{array} \end{array}$$

where $\theta_{s}$ and $\theta_{r}$ are the saturated and residual water contents, α and *n* are empirical shape parameters, and m=1−1/n. The relative hydraulic conductivity is then given by the Mualem model [9],

(104) $$\begin{array}{c} \begin{array}{c} K_{r}\left(S_{e}\right)=S_{e}^{\ell }{\left[1-{\left(1-S_{e}^{1/m}\right)}^{m}\right]}^{2}, \end{array} \end{array}$$

where *ℓ* is a pore connectivity parameter. The parameters $\left\{\alpha ,n,\theta_{s},\theta_{r},\ell \right\}$ are treated as known constants throughout the EKF inversion; they can be determined a priori from laboratory experiments or pedotransfer functions. If desired, this parameter set could also be estimated using EKF subject to some predetermined constraints on the parameter ranges.

With this parameterization, the state vector of the EKF is defined as

(105) $$\begin{array}{c} \begin{array}{c} x_{k}={\left[c_{1},c_{2},\ldots ,c_{M}\right]}^{T}\in R^{M} \end{array} \end{array}$$

and the observation operator $F(x_{k})$ maps the coefficients to the pressure head by solving the Richards Equation (96) with the conductivity field constructed from (102). Thus, the inverse problem reduces to estimating the finite-dimensional coefficient vector $x_{k}$ from head observations.

Specifically, let the state vector $x_{k}\in R^{M}$ represent the parameters to be estimated. The state evolution equation is given by

$$x_{k}=x_{k-1}+w_{k},\;w_{k}∼N\left(0,Q_{k}\right),$$

where $Q_{k}$ is the process noise covariance matrix.

The observation equation relates the state to the measured quantities through a nonlinear function F(·)

$$z_{k}=F\left(x_{k}\right)+v_{k},\;v_{k}∼N\left(0,R_{k}\right),$$

where $z_{k}\in R^{N}$ is the observation vector (e.g., pressure head measurements) and $R_{k}$ is the observation noise covariance matrix. The nonlinear function $F(x_{k})$ to $z_{k}$ is defined by the solution solver of the Richards equation; alternatively, one can learn this mapping using an operator learning method such as the DeepONet approach.

This inverse problem differs from the one discussed in the previous section in the choice of the observation variable $z_{k}$. Given the time-dependent nature of the pressure head values in unsaturated soil, we present two choices. In the first choice, we lump all available observed pressure head values at $\left(x_{j},t_{k}\right),j=n_{f}+1,\cdots ,N,k=0,\cdots ,N_{t}$ to one vector $z_{l}\in R^{n_{h}N_{t}}$, where l=1,2,⋯, is the EKF iteration index. At each time, F maps $x_{l}$ into $z_{l}$ for l=0,1,2,⋯. We hope that $x_{l}$ converges to a limit in the EKF recursion, from which the hydraulic conductivity can then be calculated. The second choice is to align the time step index in $t_{k}$ with the iteration index so that $z_{k}={\left(h\left(x_{j-n_{f}},t_{k}\right)\right)}^{T}$. This will require either the measurement to be consecutive without a limit, i.e., k=1,2,⋯. This continuous measurement is the prerequisite for the convergence of $x_{k}$. Alternatively, one must introduce a second index for internal iteration using the EKF: $z_{k}^{\left(l\right)},l=1,\cdots$. In the following, our proposed methods apply to all the above cases; however, we will use the last alternative choice as an example.

In practical applications to flows in unsaturated soil, the choice of the covariance matrices $Q_{k}$ and $R_{k}$ requires careful consideration. The process noise covariance $Q_{k}$ is typically set to a small diagonal matrix $Q_{k}=\sigma_{q}^{2}I$, where $\sigma_{q}^{2}$ is chosen to reflect the expected magnitude of unmodeled temporal variations in the log-conductivity field. A common practice is to take $\sigma_{q}^{2}$ as a fraction of the diagonal entries of the prior covariance $P_{0}^{-}$, so that the EKF iterations remain numerically stable without artificially inflating the uncertainty. The observation noise covariance $R_{k}$ should account for both measurement error and model error arising from the simplified representation of the soil–water retention curve, the finite representation of the saturated hydraulic conductivity, and the numerical solver. When pressure head observations are collected at multiple time instants, $R_{k}$ may be taken as a diagonal matrix containing entries proportional to the variance of the head measurements at each location, or as a constant multiple of the identity matrix if the measurement accuracy is uniform across sensors. In either case, the magnitude of $R_{k}$ controls the relative weight assigned to the observation term in the cost function (44) and directly influences the convergence rate and the smoothness of the reconstructed conductivity field.

#### Initialization of Expansion Coefficients Using Co-Kriging

Recall from Section 3.4 that the SLE initial estimate is obtained by co-Kriging the log-conductivity and pressure head measurements. In the present setting, the unknown quantity is the coefficient vector $c={\left[c_{1},\ldots ,c_{M}\right]}^{T}$ of the expansion. Because E[c]=0, the estimator takes the form of a co-Kriging

(106) $$\begin{array}{c} \begin{array}{c} {\hat{c}}_{0}=\Lambda^{T}f^{*}+M^{T}h^{*}, \end{array} \end{array}$$

where $f^{*}\in R^{n_{f}}$ contains the de-meaned log-conductivity measurements

$$f_{i}^{*}=\ln K_{s}^{\mathrm{obs}}\left(x_{i}\right)-F\left(x_{i}\right),\;i=1,\ldots ,n_{f}$$

and $h^{*}\in R^{n_{h}}$ contains the de-meaned pressure head measurements

$$h_{j}^{*}=\phi^{\mathrm{obs}}\left(x_{j}\right)-E\left[\phi \left(x_{j}\right)\right],\;j=1,\ldots ,n_{h}$$

where $E[\phi \left(x_{j}\right)]$ is the prior mean pressure head at $x_{j}$ obtained by solving the Richards Equation (96) with the prior mean conductivity field $K_{s}\left(x\right)=\exp\left(F\left(x\right)\right)$ and the prescribed retention curve.

In practice, the total hydraulic conductivity K(θ,x) is measured at a set of locations ${\left\{x_{i}\right\}}_{i=1}^{n_{f}}$. At these locations, the pressure head ϕ is not necessarily measured simultaneously. To extract the corresponding saturated hydraulic conductivity, the following procedure is used. First, the Richards Equation (96) is solved with the prior mean conductivity field $K_{s}\left(x\right)=\exp\left(F\left(x\right)\right)$ and the van Genuchten-Mualem model (103)–(104) to obtain the prior pressure head field $\phi^{\left(0\right)}\left(x\right)$. At each measurement location $x_{i}$, the relative conductivity is then evaluated as

$$K_{r}^{\mathrm{VG}}\left(\phi^{\left(0\right)}\left(x_{i}\right)\right)=S_{e}^{\ell }{\left[1-{\left(1-S_{e}^{1/m}\right)}^{m}\right]}^{2},$$

where $S_{e}$ is computed from $\phi^{\left(0\right)}\left(x_{i}\right)$ via (103). The corresponding saturated hydraulic conductivity is obtained as

(107) $$\begin{array}{c} \begin{array}{c} K_{s}^{\mathrm{obs}}\left(x_{i}\right)=\frac{K^{\mathrm{obs}}\left(\theta_{i},x_{i}\right)}{K_{r}^{\mathrm{VG}}\left(\phi^{\left(0\right)}\left(x_{i}\right)\right)},\;\ln K_{s}^{\mathrm{obs}}\left(x_{i}\right)=\ln K^{\mathrm{obs}}\left(\theta_{i},x_{i}\right)-\ln K_{r}^{\mathrm{VG}}\left(\phi^{\left(0\right)}\left(x_{i}\right)\right). \end{array} \end{array}$$

These $\ln K_{s}^{\mathrm{obs}}$ values are then de-meaned by subtracting the prior mean $F(x_{i})$ to obtain the fluctuation measurements $f_{i}^{*}$ used in the co-Kriging initialization. This procedure is physically consistent: the pressure head used to compute $K_{r}$ is obtained from the same forward model that governs the flow, rather than from a purely statistical interpolation. If pressure head measurements are available at the conductivity measurement locations, they can be used directly in place of $\phi^{\left(0\right)}\left(x_{i}\right)$ to avoid the dependence on the prior mean field.

The weight matrices $\Lambda \in R^{n_{f}\times M}$ and $M\in R^{n_{h}\times M}$ are obtained by minimizing the trace of the estimation error covariance $E[\left(c-{\hat{c}}_{0}\right){\left(c-{\hat{c}}_{0}\right)}^{T}]$. The resulting system is

(108) $$\begin{array}{c} \begin{array}{c} \left[\begin{array}{cc} C_{ff}^{\mathrm{obs}} & C_{fh}^{\mathrm{obs}}\\ C_{hf}^{\mathrm{obs}} & C_{hh}^{\mathrm{obs}} \end{array}\right]\left[\begin{array}{c} \Lambda \\ M \end{array}\right]=\left[\begin{array}{c} \Phi_{f}\Sigma_{c}\\ C_{ch}^{T} \end{array}\right], \end{array} \end{array}$$

where $C_{ff}^{\mathrm{obs}}\in R^{n_{f}\times n_{f}}$ is the auto-covariance matrix of the log-conductivity fluctuations at the measurement locations,

$$C_{ff}^{\mathrm{obs}}=\Phi_{f}\Sigma_{c}\Phi_{f}^{T},$$

with $\Phi_{f}\in R^{n_{f}\times M}$ defined by ${\left[\Phi_{f}\right]}_{ik}=\varphi_{k}\left(x_{i}\right)$; $C_{hh}^{\mathrm{obs}}\in R^{n_{h}\times n_{h}}$ is the auto-covariance matrix of the pressure head perturbations; and $C_{fh}^{\mathrm{obs}}\in R^{n_{f}\times n_{h}}$ is the cross-covariance matrix between the log-conductivity fluctuations and the head perturbations. $C_{fh}^{\mathrm{obs}}$ can be evaluated via first-order sensitivity as

(109) $$\begin{array}{c} \begin{array}{c} C_{fh}^{\mathrm{obs}}\approx \Phi_{f}\Sigma_{c}S^{T}, \end{array} \end{array}$$

where $S\in R^{n_{h}\times M}$ is the sensitivity matrix with entries $S_{jk}=\partial h_{j}/\partial c_{k}$. Note that this expression is consistent with $C_{ch}=\Sigma_{c}S^{T}$ below and that the relation $C_{fh}^{\mathrm{obs}}=\Phi_{f}C_{ch}$. In addition, $\Sigma_{c}\in R^{M\times M}$ is the prior covariance of the expansion coefficients, while $C_{ch}\in R^{M\times n_{h}}$ is the cross-covariance matrix between the coefficients c and the head perturbations. Its (i,j) entry is

$${\left[C_{ch}\right]}_{ij}=\mathrm{Cov}\left[c_{i},h_{j}^{*}\right]\approx \sum_{k=1}^{M}{\left[\Sigma_{c}\right]}_{ik}\;\frac{\partial h_{j}}{\partial c_{k}},$$

where $\partial h_{j}/\partial c_{k}$ is the sensitivity of the pressure head at observation point $x_{j}$ with respect to the coefficient $c_{k}$, evaluated by solving the adjoint of the discretized Richards equation.

The system (108) is solved once. The corresponding initial error covariance is

(110) $$\begin{array}{c} \begin{array}{c} P_{0,c}=\Sigma_{c}-\left[\begin{array}{cc} \Lambda^{T} & M^{T} \end{array}\right]\left[\begin{array}{cc} C_{ff}^{\mathrm{obs}} & C_{fh}^{\mathrm{obs}}\\ C_{hf}^{\mathrm{obs}} & C_{hh}^{\mathrm{obs}} \end{array}\right]\left[\begin{array}{c} \Lambda \\ M \end{array}\right]\in R^{M\times M}. \end{array} \end{array}$$

Expanding (110) gives the four-term expression

(111) $$\begin{array}{c} \begin{array}{c} P_{0,c}=\Sigma_{c}-\Lambda^{T}C_{ff}^{\mathrm{obs}}\Lambda -\Lambda^{T}C_{fh}^{\mathrm{obs}}M-M^{T}C_{hf}^{\mathrm{obs}}\Lambda -M^{T}C_{hh}^{\mathrm{obs}}M. \end{array} \end{array}$$

Equations (106) and (111) provide the initial state and error covariance for the EKF recursion described below. If the covariance of the log-conductivity field on the computational grid is needed (e.g., for visualization or uncertainty quantification), it can be recovered by a postprocessing step

(112) $$\begin{array}{c} \begin{array}{c} P_{\ln K_{s}}=\Phi \;P_{0,c}\;\Phi^{T}, \end{array} \end{array}$$

where $\Phi \in R^{N\times M}$ is the basis matrix evaluated at all grid nodes, with entries ${\left[\Phi \right]}_{ai}=\varphi_{i}\left(x_{a}\right)$.

It is worth noting that the EKF recursion could in principle be started from an uninformative prior, e.g., ${\hat{c}}_{0}=0$ and $P_{0,c}=\Sigma_{c}$, without performing the co-Kriging initialization. In that case, the initial fluctuation field would be zero everywhere and the EKF would rely entirely on the first few correction steps to adjust the coefficients toward plausible values. However, the co-Kriging-based initialization incorporates the available $\ln K_{s}$ and pressure head measurements into the initial estimate of *c* and reduces the prior covariance according to the explanatory power of these observations. This yields a starting field that is physically more consistent with the observed data and typically accelerates the convergence of the iterative estimator. The advantage is most pronounced when direct conductivity measurements are sparse, since the head observations, which are more abundant, help to constrain the initial coefficients through the cross-covariance structure encoded in $C_{fh}^{\mathrm{obs}}$ and $C_{ch}$.

At each EKF iteration, the Richards equation is solved numerically with the reconstructed $K_{s}^{\left(k\right)}\left(x\right)K_{r}\left(\theta \left(x,t\right)\right)$ in order to obtain the pressure head field $\phi^{\left(k\right)}\left(x,t\right)$. The van Genuchten–Mualem model provides the constitutive relations θ(ϕ) and $K_{r}\left(\phi \right)$, which are evaluated at the current iterate of ϕ within the nonlinear solver.

Within the EKF framework, the dependence of the observation operator F on the state vector $x_{k}$ is mediated by the parameterization in (101)–(104). At each EKF iteration, the Jacobian matrix

$$J_{k}={\left.\frac{\partial F}{\partial x}\right|}_{{\hat{x}}_{k}^{-}}\in R^{n_{h}\times M}$$

is evaluated by applying the chain rule through the Richards equation solver. Specifically, for the *i*-th component of the state vector,

(113) $$\begin{array}{c} \begin{array}{c} \frac{\partial F}{\partial c_{i}}=\frac{\partial F}{\partial K}\cdot \frac{\partial K}{\partial (\ln K_{s})}\cdot \frac{\partial (\ln K_{s})}{\partial c_{i}}=\frac{\partial F}{\partial K}\cdot K\left(\theta ,x\right)\cdot \varphi_{i}\left(x\right), \end{array} \end{array}$$

where ∂F/∂K is obtained from the adjoint of the discretized Richards Equation (96). The derivatives dθ/dϕ and ∂K/∂θ required by the adjoint solver are supplied analytically from the van Genuchten–Mualem Formulas (103)–(104). Specifically, when the Richards Equation (96) is discretized, dθ/dϕ enters through the time derivative term ∂θ/∂t and ∂K/∂θ enters through the flux term ∇·[K(θ,x)∇(ϕ+z)]; both are required by the adjoint solver when computing $J_{k}$. These analytical derivatives improve the accuracy and efficiency of the Jacobian computation compared with purely numerical differentiation.

Extending the static EKF formulation to the transient unsaturated case in the first choice of the observation variable $z_{k}$ is straightforward; however, it requires assimilating observations at multiple time instants $t_{k}$ in the second choice of $z_{k}$. Here, the iteration index *k* is aligned with the temporal index of observations, and inner/outer iterations are introduced to handle the strong nonlinearity of the Richards equation [46]. Then, the EKF prediction–correction procedure with inner iterations for the unsaturated case proceeds as follows (see Algorithm 5). Let the subscript *k* denote the time step index and let the superscript (ℓ) denote the inner iteration index within the same time step. At each time step *k*, pressure head observations $z_{k}={\left[\phi^{\mathrm{obs}}\left(x_{j},t_{k}\right)\right]}_{j=1}^{n_{h}}$ collected at time $t_{k}$ are assimilated. The observation operator $F_{k}\left(\cdot \right)$ solves the transient Richards equation over the interval $\left[t_{k-1},t_{k}\right]$ with the initial condition inherited from the previous assimilation step, and the Jacobian $J_{k}$ is evaluated at the current state estimate and time $t_{k}$.

**Algorithm** **5** (EKF-Based Parametric Inversion for Unsaturated Flow)**.**

*(1) Prediction Step. For each time step k=1,2,…,*
  (114) $$\begin{array}{cc} \mathrm{prior}\;\mathrm{state}\;\mathrm{estimate}:\;\; & {\hat{x}}_{k}^{-}={\hat{x}}_{k-1}\\ \mathrm{prior}\;\mathrm{error}\;\mathrm{covariance}:\;\; & P_{k}^{-}=P_{k-1}+Q_{k} \end{array}$$
*(2) Correction Step with inner iterations. Set ${\hat{x}}_{k}^{\left(0\right)}={\hat{x}}_{k}^{-}$; for $\ell =1,2,\ldots ,L_{\max}$, compute the Jacobian matrix of the observation function*
  $$J_{k}^{\left(\ell \right)}=\frac{\partial F_{k}}{\partial x}{|}_{{\hat{x}}_{k}^{\left(\ell -1\right)}}.$$

(115) $$\begin{array}{cc} \mathrm{Kalman}\;\mathrm{gain}:\; & K_{k}^{\left(\ell \right)}=P_{k}^{-}{\left[J_{k}^{\left(\ell \right)}\right]}^{T}{\left(J_{k}^{\left(\ell \right)}P_{k}^{-}{\left[J_{k}^{\left(\ell \right)}\right]}^{T}+R_{k}\right)}^{-1}\\ \mathrm{posterior}\;\mathrm{state}\;\mathrm{estimate}:\; & {\hat{x}}_{k}^{\left(\ell \right)}={\hat{x}}_{k}^{\left(\ell -1\right)}+K_{k}^{\left(\ell \right)}\left(z_{k}-F_{k}\left({\hat{x}}_{k}^{\left(\ell -1\right)}\right)\right)\\ \mathrm{posterior}\;\mathrm{error}\;\mathrm{covariance}:\; & P_{k}^{\left(\ell \right)}=\left(I-K_{k}^{\left(\ell \right)}J_{k}^{\left(\ell \right)}\right)P_{k}^{-} \end{array}$$

*At each inner iteration, the current $\phi^{\left(\ell \right)}$ is used to evaluate $K_{r}\left(\phi \right)$ via (103)–(104), so that $K=K_{s}\cdot K_{r}\left(\phi \right)$ is self-consistently updated within the nonlinear solver.*

*The inner iteration terminates when*

(116) $$\begin{array}{c} \begin{array}{c} \frac{∥{\hat{x}}_{k}^{\left(\ell \right)}-{\hat{x}}_{k}^{\left(\ell -1\right)}{∥}_{2}}{∥{\hat{x}}_{k}^{\left(\ell -1\right)}{∥}_{2}}<\varepsilon_{\mathrm{inner}} \end{array} \end{array}$$

*or when $\ell =L_{\max}$. Denote by L ($L\leq L_{\max}$) the actual number of inner iterations executed before the stopping criterion (116) is met. Then, the posterior estimate and covariance at time step k are*

(117) $$\begin{array}{c} \begin{array}{c} {\hat{x}}_{k}={\hat{x}}_{k}^{\left(L\right)},\;P_{k}=P_{k}^{\left(L\right)}. \end{array} \end{array}$$

*In practice, to balance accuracy and computational cost, $J_{k}^{\left(\ell \right)}$ may be recomputed only when the relative change of ${\hat{x}}_{k}^{\left(\ell \right)}$ exceeds a threshold.*

*Because the observation operator $F_{k}$ defined by the transient Richards equation is strongly nonlinear, a single sequential pass over $k=1,\ldots ,N_{t}$ may terminate in a locally linearized solution. To mitigate this, it is possible to perform multiple passes over the same spatiotemporal dataset, with each pass using the posterior of the previous pass as the prior until the estimate stabilizes.*

*(3) Stopping criterion for the outer loop. Let ${\hat{x}}_{N_{t}}^{\left(p\right)}$ denote the posterior estimate of the state vector x after the p-th complete pass over all $N_{t}$ time steps. In each pass, the EKF proceeds sequentially from k=1 to $k=N_{t}$ according to the prediction–correction procedure described above, and ${\hat{x}}_{N_{t}}^{\left(p\right)}$ is the final estimate produced at the end of the p-th pass. Therefore, the subscript $N_{t}$ indicates that the estimate is obtained after assimilating the entire set of spatiotemporal observations, not merely the observation at the single time instant $t_{N_{t}}$.*

*The outer loop is terminated when the relative change between two consecutive passes falls below a prescribed tolerance*

(118) $$\begin{array}{c} \begin{array}{c} \frac{∥{\hat{x}}_{N_{t}}^{\left(p\right)}-{\hat{x}}_{N_{t}}^{\left(p-1\right)}{∥}_{2}}{∥{\hat{x}}_{N_{t}}^{\left(p-1\right)}{∥}_{2}}<\varepsilon_{\mathrm{outer}} \end{array} \end{array}$$

*or when a preset maximum number of passes $P_{\max}$ is reached. The first pass (p=1) starts from the co-Kriging initialization ${\hat{x}}_{0}={\hat{c}}_{0}$ and $P_{0}=P_{0,c}$, then proceeds through $k=1,\ldots ,N_{t}$ to produce ${\hat{x}}_{N_{t}}^{\left(1\right)}$. A typical choice for the inner tolerance is $\varepsilon_{\mathrm{inner}}=10^{-3}$ to $10^{-4}$, with a maximum of $L_{\max}=5$*–10* inner iterations per time step. The outer-loop tolerance is set to the same range, $\varepsilon_{\mathrm{outer}}=10^{-3}$ to $10^{-4}$. These values are standard for iterative EKF schemes applied to nonlinear inverse problems governed by the Richards equation [46]. The inner criterion controls convergence of the local linearized update at each time step, while the outer criterion assesses global stability across the entire assimilation pass. In practice, relative changes below $10^{-3}$ already correspond to negligible adjustments of the estimated $\ln K_{s}$ field and the associated hydraulic-head predictions. Together, the two criteria ensure that the estimated field has stabilized under repeated assimilation of the same dataset, thereby mitigating the effect of the local linearization inherent in the EKF and promoting convergence toward a consistent solution.*

### 4.2. Data-Driven Parameter Estimation for Unsaturated Flow Using the Ensemble Kalman Filter

The preceding sections have focused on forward modeling under the assumption that the hydraulic conductivity follows prescribed parametric forms. In practice, however, the van Genuchten–Mualem model, while widely used, imposes a rigid relationship between the hydraulic conductivity *K* and the pressure head ϕ that may not hold in heterogeneous or structured soils. A purely parametric inversion that treats the VGM parameters as unknown still inherits this structural constraint, and the estimated *K* field is forced into a specific functional shape that may deviate from the true behavior.

An alternative is to abandon any parametric assumptions and learn K(x,ϕ) directly from data. This is appealing in its generality, but introduces severe ill-posedness because the unknown K(x,ϕ) lies in a high-dimensional space indexed by both spatial position and pressure head, while the available observations are far too sparse to constrain it uniquely. Without additional structure, the inversion yields a highly non-unique solution with little physical interpretability.

A pragmatic compromise is to retain the physically interpretable saturation curve θ(ϕ), which can be measured independently in the laboratory, while relaxing the VGM constraint on the conductivity. This decomposition is not merely a matter of convenience. A direct Karhunen–Loève expansion of K(x,ϕ) as a single field would require a two-dimensional covariance kernel indexed by both spatial location and pressure head, substantially increasing the number of modes needed to capture the full variability; moreover, such an approach would discard the strong physical prior provided by the van Genuchten–Mualem model, which prescribes a well-established functional relationship between $K_{r}$ and ϕ. The spatial variability of $K_{s}\left(x\right)$ and the functional dependence of $K_{r}\left(\phi \right)$ operate on different physical scales and carry very different prior knowledge: $K_{s}\left(x\right)$ is a stationary or quasi-stationary random field amenable to geostatistical modeling, while $K_{r}\left(\phi \right)$ is a bounded monotone function with a shape that is well approximated by the van Genuchten–Mualem model. Merging them into a single object discards this structural distinction and forces the inversion to learn both aspects from the same limited data, which is neither efficient nor robust.

By separating the two factors, we retain the VGM prediction as a physically interpretable baseline for the relative hydraulic conductivity and restrict the KL expansion to the deviation from this baseline. The saturated conductivity $K_{s}\left(x\right)$, which depends only on spatial position and is already well studied in hydrogeology, is estimated directly on the full grid using the ensemble approximation. In this way, the total number of degrees of freedom is kept moderate, the physical structure of unsaturated flow is preserved, and the data-driven correction is confined to the component that is least constrained by existing theory. Specifically, we decompose the hydraulic conductivity as

(119) $$\begin{array}{c} \begin{array}{c} K\left(x,\phi \right)=K_{s}\left(x\right)\;K_{r}\left(x,\phi \right), \end{array} \end{array}$$

where $K_{s}\left(x\right)$ is the saturated hydraulic conductivity and $K_{r}\left(x,\phi \right)$ is the relative hydraulic conductivity function. The saturated conductivity $K_{s}\left(x\right)$ is unknown and must be inferred from data. For the relative hydraulic conductivity, we treat the VGM prediction as a prior mean but allow spatially varying deviations. This is achieved by expanding $K_{r}$ in a Karhunen–Loève basis,

(120) $$\begin{array}{c} \begin{array}{c} K_{r}\left(x,\phi \right)=K_{r}^{\mathrm{VG}}\left(\phi \right)+\sum_{i=1}^{m}\sqrt{\lambda_{i}}\;\alpha_{i}\left(x\right)\;\psi_{i}\left(\phi \right), \end{array} \end{array}$$

where ${\left\{\lambda_{i},\psi_{i}\left(\phi \right)\right\}}_{i=1}^{m}$ are the leading eigenvalues and eigenfunctions of the covariance kernel of $K_{r}$ sampled from a prior ensemble and where $\alpha_{i}\left(x\right)$ are spatially correlated coefficient fields to be estimated. The number of modes *m* is chosen such that the expansion captures a prescribed fraction of the prior variance. This construction respects the VGM as a baseline while allowing data-driven corrections that are spatially heterogeneous and physically plausible.

The covariance kernel for the deviation of $K_{r}$ from the VGM baseline is prescribed as a stationary exponential form

(121) $$\begin{array}{c} \begin{array}{c} C_{K_{r}}\left(\phi ,\phi^{\prime }\right)=\sigma_{K_{r}}^{2}\exp\left(-\frac{|\phi -\phi^{\prime }|}{l_{K_{r}}}\right), \end{array} \end{array}$$

where $\sigma_{K_{r}}^{2}$ denotes the prior variance and $l_{K_{r}}$ the correlation length in the pressure head space. This choice follows standard geostatistical practice for deriving KL expansions in hydrogeological parameter fields [6] and ensures positive definiteness and smoothness consistent with laboratory retention measurements.

The eigenvalues $\lambda_{i}$ and eigenfunctions $\psi_{i}\left(\phi \right)$ are obtained by solving the eigendecomposition of a prescribed covariance kernel for the deviation of $K_{r}$ from the VGM baseline, either analytically from a chosen kernel $C_{K_{r}}\left(\phi ,\phi^{\prime }\right)$ or empirically from a prior ensemble of plausible $K_{r}\left(\phi \right)$ curves. This decomposition is performed before the assimilation and the resulting $\psi_{i}\left(\phi \right)$ are held fixed thereafter; they serve as a fixed functional basis on the ϕ-axis along which the spatially varying correction $\alpha_{i}\left(x\right)$ acts. Thus, the temporal variation of $K_{r}\left(x,\phi \left(x,t\right)\right)$ arises solely from the transient ϕ(x,t) passed through these fixed $\psi_{i}$ and the fixed VGM function $K_{r}^{\mathrm{VG}}\left(\phi \right)$.

Unlike the EKF formulation in Section 4.1, where the state dimension must be kept small because the Jacobian matrix and the full covariance matrix must be computed and stored explicitly, the EnKF circumvents these requirements by approximating the state covariance through an ensemble of realizations. Consequently, $\ln K_{s}\left(x\right)$ can be estimated directly on the full computational grid without a separate KL expansion. This removes the need to impose a low-dimensional representation on the saturated conductivity field. Instead, the flexibility gained from the ensemble approximation is allocated to the relative hydraulic conductivity $K_{r}\left(x,\phi \right)$, which is allowed to deviate from the VGM baseline through a KL expansion. In this way, the saturated conductivity retains its full spatial degrees of freedom, while the unsaturated conductivity benefits from a data-driven correction that is both parsimonious and physically constrained.

The relative hydraulic conductivity $K_{r}\left(x,\phi \right)$ is required to be non-decreasing in ϕ and to satisfy $0<K_{r}\left(x,\phi \right)\leq 1$, with $K_{r}\left(x,0\right)=1$. To enforce this physically motivated constraint within the ensemble Kalman filter, the Karhunen–Loève basis $\left\{\psi_{i}\left(\phi \right)\right\}$ is constructed from a prior ensemble restricted to monotone $K_{r}$ curves consistent with the van Genuchten retention model. In addition, after each analysis step, ensemble members for which the reconstructed $K_{r}\left(x,\phi \right)$ violates monotonicity or falls outside (0,1] are projected back onto the nearest admissible curve by monotone isotonic correction and clipping. The saturated conductivity is handled in log-space and clipped to a physically reasonable range. These procedures ensure that all realizations entering the forward Richards solver remain thermodynamically consistent.

The unknown parameter field now consists of $\ln K_{s}\left(x\right)$ and the *m* coefficient fields ${\left\{\alpha_{i}\left(x\right)\right\}}_{i=1}^{m}$. The total number of degrees of freedom is $\left(1+m\right)N_{x}$, where $N_{x}$ is the number of spatial grid points. Even for moderate *m* and a modest spatial discretization, the state dimension easily exceeds several thousand. This rules out methods that require explicit computation of the Jacobian, such as the extended Kalman filter, which would demand $O(N_{s})$ forward solves per assimilation step and the storage of an $N_{s}\times N_{s}$ covariance matrix.

The ensemble Kalman filter offers a natural alternative. It approximates the state covariance using an ensemble of $N_{e}$ realizations, requiring only $N_{e}$ forward solves per step—typically two orders of magnitude fewer than the state dimension. No tangent-linear or adjoint model is needed, making the method agnostic to the complexity of the forward solver. The ensemble representation also captures nonlinear propagation of uncertainty without linearization, which is critical for the strongly nonlinear Richards equation. For these reasons, the EnKF is adopted as the inference engine in this work.

#### 4.2.1. State Vector and Observation Operator

Let the computational domain be discretized into $N_{x}$ grid cells with grid points $x_{1},\cdots ,x_{N_{x}}$. The state vector at assimilation step *k* is defined as

(122) $$\begin{array}{c} \begin{array}{c} m_{k}={\left[\ln K_{s}\left(x_{1}\right),\ldots ,\ln K_{s}\left(x_{N_{x}}\right),\;\alpha_{1}\left(x_{1}\right),\ldots ,\alpha_{1}\left(x_{N_{x}}\right),\;\ldots ,\;\alpha_{m}\left(x_{1}\right),\ldots ,\alpha_{m}\left(x_{N_{x}}\right)\right]}^{T}\in R^{N_{s}}, \end{array} \end{array}$$

where $N_{s}=\left(1+m\right)N_{x}$. The logarithmic transformation ensures positivity of $K_{s}$ and improves the Gaussianity of the prior.

Given a state realization m, the hydraulic conductivity field is reconstructed as

(123) $$\begin{array}{c} \begin{array}{c} K\left(x,\phi \right)=\exp\left(\ln K_{s}\left(x\right)\right)\left[K_{r}^{\mathrm{VG}}\left(\phi \right)+\sum_{i=1}^{m}\sqrt{\lambda_{i}}\alpha_{i}\left(x\right)\;\psi_{i}\left(\phi \right)\right] \end{array} \end{array}$$

and the water retention curve θ(ϕ) is taken from the independently calibrated VGM. Substituting these into the Richards equation and solving numerically yields the predicted pressure head field ϕ(x,t). The observation operator H extracts the predicted equivalents of the measurements. Let $N_{\mathrm{obs}}^{\mathrm{loc}}$ denote the number of observation locations and $N_{t}$ the number of assimilation time steps. The total number of observations is $N_{\mathrm{obs}}=N_{\mathrm{obs}}^{\mathrm{loc}}\times N_{t}$. Each observation location $x_{j}^{\mathrm{obs}}$ coincides with a computational grid node, so that the observation operator H simply extracts the simulated pressure head at the corresponding grid point. The observation vector is then

(124) $$\begin{array}{c} \begin{array}{c} H\left(m\right)={\left[\phi \left(x_{1}^{\mathrm{obs}},t_{1}\right),\ldots ,\phi \left(x_{N_{\mathrm{obs}}^{\mathrm{loc}}}^{\mathrm{obs}},t_{1}\right),\ldots ,\phi \left(x_{1}^{\mathrm{obs}},t_{N_{t}}\right),\ldots ,\phi \left(x_{N_{\mathrm{obs}}^{\mathrm{loc}}}^{\mathrm{obs}},t_{N_{t}}\right)\right]}^{T}\in R^{N_{\mathrm{obs}}}. \end{array} \end{array}$$

Measurements may include pressure heads from tensiometers or water content readings from soil moisture sensors; in the latter case, the conversion θ=θ(ϕ) is applied before comparison.

#### 4.2.2. Iterative EnKF with Parameter Localization

The ensemble Kalman filter is implemented in a standard form with covariance localization to mitigate spurious long-range correlations arising from finite ensemble size [39].

At initialization, draw $N_{e}$ samples ${\left\{m_{0}^{\left(j\right)}\right\}}_{j=1}^{N_{e}}$ from the prior distribution. For $\ln K_{s}$, a Gaussian random field with exponential covariance is used. In practice, the variance and correlation length of the exponential covariance are estimated from available measurements of $\ln K_{s}$, for example by variogram fitting or selected based on prior geological knowledge when data are limited. These parameters are problem-dependent; for the KL coefficients, the prior is $\alpha_{i}∼N\left(0,\lambda_{i}\right)$, where $\lambda_{i}$ is the eigenvalue associated with $\psi_{i}$. The iterative EnKF with parameter localization is summarized in Algorithm 6.

**Algorithm** **6** (Iterative EnKF with Parameter Localization)**.**

*(1) Forecast step. For each ensemble member j, solve the Richards equation over the assimilation window $\left[t_{k-1},t_{k}\right]$ using the reconstructed $K^{\left(j\right)}\left(x,\phi \right)$ and the fixed θ(ϕ). Collect the predicted observations*
(125) $$\begin{array}{c} \begin{array}{c} y_{k}^{\left(j\right)}=H_{k}\left(m_{k-1}^{\left(j\right)}\right)+\epsilon_{k}^{\left(j\right)},\;\epsilon_{k}^{\left(j\right)}∼N\left(0,R_{k}\right), \end{array} \end{array}$$
  *where $R_{k}$ is the observation error covariance and the perturbation avoids ensemble collapse.*
*(2) Analysis step. Compute the ensemble mean and covariance*
(126) $$\begin{array}{c} \begin{array}{c} P_{my}=\frac{1}{N_{e}-1}\sum_{j=1}^{N_{e}}\left(m_{k-1}^{\left(j\right)}-{\bar{m}}_{k}^{-}\right){\left(y_{k}^{\left(j\right)}-{\bar{y}}_{k}^{-}\right)}^{T},\;P_{yy}=\frac{1}{N_{e}-1}\sum_{j=1}^{N_{e}}\left(y_{k}^{\left(j\right)}-{\bar{y}}_{k}^{-}\right){\left(y_{k}^{\left(j\right)}-{\bar{y}}_{k}^{-}\right)}^{T}, \end{array} \end{array}$$
  *where*
(127) $$\begin{array}{c} \begin{array}{c} {\bar{m}}_{k}^{-}=\frac{1}{N_{e}}\sum_{j=1}^{N_{e}}m_{k-1}^{\left(j\right)},\;{\bar{y}}_{k}^{-}=\frac{1}{N_{e}}\sum_{j=1}^{N_{e}}y_{k}^{\left(j\right)}. \end{array} \end{array}$$
  *Apply covariance localization by Schur product with a compactly supported correlation matrix C,*
(128) $$\begin{array}{c} \begin{array}{c} P_{my}^{\mathrm{loc}}=C\circ P_{my},\;P_{yy}^{\mathrm{loc}}=C_{\mathrm{obs}}\circ P_{yy}. \end{array} \end{array}$$
  *The matrix C in the Hadamard product $C\circ P_{my}$ is a compactly supported correlation matrix that implements covariance localization. Its entry $C_{ij}$ encodes the presumed correlation between the i-th state variable and the j-th observation, and is defined as a function of their spatial separation*
$$C_{ij}=\rho \;\left(\frac{∥x_{i}^{\mathrm{state}}-x_{j}^{\mathrm{obs}}∥}{L}\right),$$
  *where $x_{i}^{\mathrm{state}}$ is the spatial coordinate associated with the i-th component of the state vector, $x_{j}^{\mathrm{obs}}$ is the location of the j-th observation, L is the localization radius, and ρ(·) is a positive definite function with compact support [47,48,49]. Similarly, $C_{\mathrm{obs}}$ is defined for observation–observation pairs and enters the Hadamard product with $P_{yy}$.*

*The purpose of localization is to suppress spurious long-range correlations that arise from sampling error when the ensemble size is much smaller than the state dimension. Without localization, the sample cross-covariance $P_{my}$ contains non-negligible entries between distant locations, causing the analysis to update parameters using observations that are physically unrelated. The Hadamard product attenuates these entries proportionally to distance, retaining only those correlations that are physically plausible. This operation is standard in ensemble-based data assimilation for high-dimensional geophysical problems [48,49].*

*The Kalman gain is then given by*

(129) $$\begin{array}{c} \begin{array}{c} K_{k}=P_{my}^{\mathrm{loc}}{\left(P_{yy}^{\mathrm{loc}}+R_{k}\right)}^{-1}. \end{array} \end{array}$$

*(3) Update each ensemble member.*
(130) $$\begin{array}{c} \begin{array}{c} m_{k}^{\left(j\right)}=m_{k-1}^{\left(j\right)}+K_{k}\left(y_{k}^{\mathrm{obs}}-y_{k}^{\left(j\right)}\right) \end{array} \end{array}$$
*(4) Inflation. To prevent underestimation of spread, multiplicative inflation is applied after each analysis:*
$$m_{k}^{\left(j\right)}\leftarrow {\bar{m}}_{k}+\gamma \left(m_{k}^{\left(j\right)}-{\bar{m}}_{k}\right)$$
  *with γ∈[1,1.05] tuned heuristically [41,50].*
*(5) Iteration. Advance to the next assimilation window and repeat until all observations have been assimilated.*

The assimilation proceeds sequentially over disjoint time windows $\left[t_{k-1},t_{k}\right]$, $k=1,\ldots ,N_{t}$. Within each window, the forward model is integrated from the initial condition inherited from the previous analysis step using the most recently updated parameter fields. At the end of the window, the EnKF analysis updates both the parameter fields and the state variables, which then serve as the initial condition for the next window. This procedure assumes that the hydraulic parameters are time-invariant over the assimilation period, which is standard for unsaturated zone inverse problems.

The EnKF simultaneously updates the $\ln K_{s}$ field and the KL coefficient fields, thereby learning a spatially heterogeneous relative hydraulic conductivity that can deviate from the VGM prediction wherever the data demand it. The KL truncation level *m* controls the flexibility of the model and is determined prior to inversion based on the prior spectrum.

#### 4.2.3. Synthetic Experiments

As this paper is theoretical in scope, we do not present numerical results here; however, to demonstrate the practical viability of the proposed framework, we outline a possible numerical validation strategy. To validate the proposed EnKF-based inversion strategy, synthetic experiments would be conducted. A reference parameter field, denoted by $K_{\mathrm{true}}\left(x,\phi \right)$, is first prescribed, then the forward Richards solver is invoked to generate the corresponding pressure head field $\phi_{\mathrm{true}}\left(x,t\right)$. Observations are extracted at selected locations and time instants, then corrupted with additive Gaussian noise to mimic measurement errors:

$$y_{j}^{\mathrm{obs}}=\phi_{\mathrm{true}}\left(x_{j}^{\mathrm{obs}},t_{k}\right)+\epsilon_{j},\;\epsilon_{j}∼N\left(0,\sigma_{\mathrm{obs}}^{2}\right).$$

The EnKF procedure described in Section 4.2.2 is applied to estimate $\ln K_{s}\left(x\right)$ and ${\left\{\alpha_{i}\left(x\right)\right\}}_{i=1}^{m}$ from these synthetic observations. The quality of the inversion is assessed by comparing the estimated fields against the reference via the root-mean-square error (RMSE) and correlation coefficient. This workflow allows for a controlled evaluation of the inversion performance under various scenarios, including different observation densities, noise levels, and prior specifications. A full numerical evaluation of the proposed framework, including synthetic and field-scale experiments, is deferred to future work.

## 5. Conclusions

### 5.1. Concluding Remarks

We have established a unified framework linking the successive linear estimator (SLE) and the extended Kalman filter (EKF) for hydraulic conductivity estimation in groundwater flow. Starting from the least-squares formulation of the Kalman filter, we show that the SLE update, traditionally derived from geostatistical principles, can be reinterpreted as an EKF correction step applied to a static parameter estimation problem, with the co-Kriging estimate supplying the initial prior and the sensitivity matrix serving as the linearized observation operator. This equivalence places the SLE within the broader context of recursive Bayesian estimation and provides a rigorous foundation for its extension beyond the saturated steady-state setting.

Building on this equivalence, we have extended the EKF framework to unsaturated flow governed by the transient Richards equation. Two complementary strategies are developed. The first strategy employs a low-dimensional basis expansion for the saturated conductivity field and applies an iterated EKF with inner–outer loop convergence criteria to assimilate spatiotemporal head observations. The second strategy adopts the ensemble Kalman filter (EnKF) to circumvent the explicit Jacobian computation required by the EKF, enabling direct estimation of the saturated conductivity on the full computational grid while representing the relative hydraulic conductivity through a KL expansion that allows data-driven deviations from the van Genuchten–Mualem baseline. Covariance localization and multiplicative inflation are incorporated to mitigate sampling errors inherent in finite-size ensembles.

### 5.2. Limitations and Future Work

Several aspects merit scrutiny before field-scale deployment. The EKF linearizes the Richards solver around the current estimate; in strongly nonlinear regimes such as sharp wetting fronts, neglected higher-order terms may bias the posterior or impede convergence, especially when the initial co-Kriging estimate is poor. Iterated EKF relinearizes at each inner loop at a cost proportional to grid size and iteration count. The equivalence between EKF and SLE implies that the iterative SLE inherits the same linearization error, and explicit matrix assembly becomes prohibitive for high-resolution three-dimensional problems. The EnKF in Section 4.2 avoids Jacobian assembly, but requires large ensembles for accurate covariance representation; localization and inflation parameters are typically calibrated empirically, and adaptive schemes would reduce this tuning burden. On the constitutive side, fixing the van Genuchten–Mualem shape parameters a priori may introduce errors when the assumed functional form mismatches reality, degrading interpretability. The EnKF strategy relaxes this via a KL expansion of relative hydraulic conductivity, yet the expansion depends on a prescribed covariance kernel for which the parameters remain uncertain. Joint estimation of shape parameters or a non-parametric hierarchical Bayesian formulation is a natural next step.

Identifiability is further limited by reliance on hydraulic head measurements, which provide weak sensitivity near saturation and carry less information under steady state than transient conditions. The extension to anisotropic (tensor) conductivity would further increase the number of unknowns, and consequently require more observations to constrain the inversion. Complementary data such as soil moisture, infiltration rates, or geophysical measurements would tighten the inverse constraint. A detailed discussion on the observability limits and the role of flux measurements is provided in Appendix A. More broadly, the theoretical developments rest on idealized boundary conditions, covariance structures, and observation configurations; real-world application introduces measurement noise, boundary misspecification, unresolved geology, and sub-grid heterogeneity. Controlled field experiments with independent characterization data, along with numerical benchmarks probing sensitivity to nonlinearity, constitute the principal directions of ongoing work. Comprehensive numerical and field-scale validation of the unified framework is reserved for future dedicated work, as the scope of the present study is restricted to theoretical derivation and methodological unification.

## Figures and Tables

**Table 1:** Comparison of Section 3 and Section 4 formulations.

	Section 3	Section 4
Flow type	Steady-state saturated flow	Transient unsaturated flow
Governing equation	∇·(K(x)∇ϕ(x))=S(x)	$\frac{\partial \theta (\phi (x,t))}{\partial t}+S\left(x,t\right)=\nabla \cdot \left[K\left(\theta ,x\right)\nabla \left(\phi (x,t)+z\right)\right]$
Primary unknown	lnK(x)	Section 4.1: $\ln K_{s}\left(x\right)$; Section 4.2: $\ln K_{s}\left(x\right)$ and $K_{r}\left(x,\phi \right)$
Parameterization	lnK(x)=F(x)+f(x)	Section 4.1: $\ln K_{s}\left(x\right)=F\left(x\right)+\sum_{i=1}^{M}c_{i}\;\varphi_{i}\left(x\right);$ Section 4.2: $K_{r}\left(x,\phi \right)=K_{r}^{\mathrm{VG}}\left(\phi \right)+\sum_{i=1}^{m}\sqrt{\lambda_{i}}\;\alpha_{i}\left(x\right)\;\psi_{i}\left(\phi \right)$
State vector	$x_{k}={\left[\ln K\left(x_{1}\right),\ldots ,\ln K\left(x_{N}\right)\right]}^{T}$	Section 4.1: $x_{k}={\left[c_{1},c_{2},\ldots ,c_{M}\right]}^{T}$ Section 4.2: $m_{k}=[\ln K_{s}\left(x_{1}\right),\ldots ,\ln K_{s}\left(x_{N_{x}}\right),\;$ $\alpha_{1}\left(x_{1}\right),\ldots ,\alpha_{1}\left(x_{N_{x}}\right),\;\ldots ,\;\alpha_{m}\left(x_{1}\right),\ldots ,\alpha_{m}\left(x_{N_{x}}\right){]}^{T}$
Observation type	Steady hydraulic head ϕ(x)	Transient pressure head ϕ(x,t)
Forward solver	Elliptic steady-state solver	Time-marching transient solver
Inversion method	SLE (equivalent to EKF)	Parametric EKF or EnKF

## Data Availability

The original contributions presented in the study are included in the article, further inquiries can be directed to the corresponding author.

## Appendix A. Discussion on Observable Quantities for Unsaturated Zone Inversion

It should be noted that the inversion of K(x,ϕ) from pressure head observations alone remains ill-posed even with the KL regularization [51,52,53]. Additional information such as flux measurements (e.g., infiltration rates, evaporation fluxes, or drainage outflow) can significantly reduce the non-uniqueness [54,55,56]. In the laboratory, fluxes are routinely measurable via lysimeters or flow meters. In the field, point-scale flux measurements are possible with permeameters, while catchment-scale fluxes can be estimated from streamflow records or baseflow separation techniques [57,58,59]. In numerical experiments, synthetic flux data can be generated to assess their impact on the inversion quality, but the practical feasibility of flux measurements in the field depends on the spatial scale and site accessibility.

While numerical solvers can generate arbitrarily dense observations for testing purposes, practical field campaigns are limited to measurements that are technically feasible and economically viable. Typical measurable quantities include pressure head (tensiometers), water content (time-domain reflectometry, neutron probes), and occasionally matric potential (heat dissipation sensors). Direct flux measurements remain challenging at the field scale [60].
